# DEPDC5 protects CD8^+^ T cells from ferroptosis by limiting mTORC1-mediated purine catabolism

**DOI:** 10.1038/s41421-024-00682-z

**Published:** 2024-05-20

**Authors:** Song Li, Xinxing Ouyang, Hongxiang Sun, Jingsi Jin, Yao Chen, Liang Li, Qijun Wang, Yingzhong He, Jiwen Wang, Tongxin Chen, Qing Zhong, Yinming Liang, Philippe Pierre, Qiang Zou, Youqiong Ye, Bing Su

**Affiliations:** 1 Shanghai Institute of Immunology, Department of Immunology and Microbiology at Basic Medical College, Shanghai Jiao Tong University School of Medicine, Shanghai, China; 2 Department of Gastroenterology and Center for Immune-Related Diseases Research at Ruijin Hospital affiliated to Shanghai Jiao Tong University School of Medicine, Shanghai, China; 3 Shanghai Chest Hospital affiliated to Shanghai Jiao Tong University School of Medicine, Shanghai, China; 4 Department of Neurology of Shanghai Children’s Medical Center affiliated to Shanghai Jiao Tong University School of Medicine, Shanghai, China; 5 Department of Allergy and Immunology, Division of Immunology and Multidisciplinary Specialty Clinic, Institute of Pediatric Translational Medicine at Shanghai Children’s Medical Center affiliated to Shanghai Jiao Tong University School of Medicine, Shanghai, China; 6 Key Laboratory of Cell Differentiation and Apoptosis of Chinese Ministry of Education, Department of Pathophysiology, Shanghai Jiao Tong University School of Medicine, Shanghai, China; 7 School of Laboratory Medicine, Xinxiang Medical University, Xinxiang, Henan China; 8 Aix Marseille Université, CNRS, INSERM, CIML, Marseille, cedex 9 France; 9 Institute of Biomedicine (iBiMED), Department of Medical Sciences, University of Aveiro, Aveiro, Portugal; 10 Shanghai Jiao Tong University School of Medicine-Yale Institute for Immune Metabolism, Shanghai Jiao Tong University School of Medicine, Shanghai, China; 11 Key Laboratory of Molecular Radiation Oncology of Hunan Province, Xiangya Hospital, Central South University, Changsha, China

**Keywords:** Cell death, Tumour immunology, Nutrient signalling

## Abstract

Peripheral CD8^+^ T cell number is tightly controlled but the precise molecular mechanism regulating this process is still not fully understood. In this study, we found that epilepsy patients with loss of function mutation of *DEPDC5* had reduced peripheral CD8^+^ T cells, and DEPDC5 expression positively correlated with tumor-infiltrating CD8^+^ T cells as well as overall cancer patient survival, indicating that DEPDC5 may control peripheral CD8^+^ T cell homeostasis. Significantly, mice with T cell-specific *Depdc5* deletion also had reduced peripheral CD8^+^ T cells and impaired anti-tumor immunity. Mechanistically, *Depdc5*-deficient CD8^+^ T cells produced high levels of xanthine oxidase and lipid ROS due to hyper-mTORC1-induced expression of ATF4, leading to spontaneous ferroptosis. Together, our study links DEPDC5*-*mediated mTORC1 signaling with CD8^+^ T cell protection from ferroptosis, thereby revealing a novel strategy for enhancing anti-tumor immunity via suppression of ferroptosis.

## Introduction

Maintaining sufficient peripheral CD8^+^ T cell number is crucial for mounting a robust host immune response against pathogen infection and the effectiveness of an anti-tumor immune response also positively correlates with the numbers of functional CD8^+^ T cells infiltrated in the tumor microenvironment^1–3^. Peripheral T cell number is tightly regulated by homeostatic mechanisms in order to keep total population size relatively constant throughout adult life^4^. This process requires MHC I recognition and cytokines like IL-7 to sustain the survival from apoptosis and homeostatic turnover of CD8^+^ T cells^5,6^. However, whether these homeostatic mechanisms intersect with the T cell metabolic pathway as well as other types of cell death remains poorly defined.

The mechanistic target of rapamycin (mTOR) is an evolutionarily conserved protein kinase that critically regulates cell growth, proliferation, and metabolism^7–9^. Activation of mTOR complex 1 (mTORC1) is inhibited by Gap Activity TOward Rags (GATOR) 1 when supplies of amino acids or glucose are limited^10–12^. *DEPDC5*, a known epilepsy risk gene encoding DEP domain-containing protein (DEPDC) 5^13–15^, is an essential component of the GATOR1 that also contains NPRL2 and NPRL3^10^. Activated mTORC1 phosphorylates the translational regulators S6K and 4E-BP1, leading to increased ribosome biogenesis and protein translation via up-regulation of multiple metabolic pathways^9^. Consequently, mTORC1-mediated metabolism generates a spectrum of intermediate compounds, many of which have been implicated in a newly identified form of programmed cell death termed ferroptosis^16,17^.

Ferroptosis is a form of regulated cell death triggered by iron-dependent oxidization of polyunsaturated fatty acids (PUFA) and accumulation of lethal levels of lipid hydroperoxides (L-OOH)^18,19^. Oxidized polyunsaturated fatty acids (ox-PUFA) are the best-known product of iron-dependent lipid peroxidation known to be associated with ferroptosis. Production of ox-PUFA requires the generation of hydroxyl radicals (OH^.^) from hydrogen peroxides (H_2_O_2_) and ferrous iron (Fe^2+^) via a process known as the Fenton reaction^20^.

Under physiological conditions, ferroptosis is inhibited by the lipid repair system which employs glutathione and glutathione peroxidase 4 (GPX4) as well as CoQ10 and ferroptosis suppressor protein 1 (FSP1) to convert L-OOH into non-toxic lipid alcohols (L-OH)^16^. Ferroptosis can also be inhibited using lipophilic antioxidants including ferrostatin-1 (Fer-1) and liproxstatin-1 (Lip-1), or with iron chelators such as deferoxamine (DFO)^21^. Vitamin E, a well-known ROS scavenger, has also been shown to effectively suppress ferroptosis in vivo and in vitro indicating that free radicals are one of the most important triggers of ferroptosis^19,22,23^.

Selective induction of cancer cell ferroptosis has been proposed as a potential strategy for anti-tumor therapy ^24–26^. For example, the depletion of extracellular cysteine strongly induced ferroptosis of pancreatic ductal adenocarcinoma (PDAC) tumors in mice and extended overall survival^26^. However, several recent studies have shown that ferroptosis in CD8^+^ T cells can also impair anti-tumor immunity ^27,28^. An early study showed that T cell-specific *Gpx4* deletion enhances ferroptosis of the CD8^+^ population^23^. In a more recent study, CD8^+^ T cells were observed to be more sensitive to ferroptosis than tumor cells in vitro^29^. Given that CD8^+^ T cell number is crucial for robust host immunity, it is important to understand the molecular mechanisms that control this population in vivo.

In this study, we showed that epilepsy patients with loss of function *DEPDC5* mutations had reduced peripheral CD8^+^ T cells whereas high DEPDC5 expression positively correlated with tumor infiltration of CD8^+^ T cells as well as overall cancer patient survival. Using a T cell-specific *Depdc5* conditional knockout mouse model, we demonstrate that DEPDC5 protects CD8^+^ T cells from ferroptosis and is required for CD8^+^ T cell-mediated anti-tumor immunity. At the molecular level, DEPDC5 deficiency causes hyper-mTORC1 activity to induce ATF4 expression, which in turn, augments the expression of xanthine oxidase (XO) and lipid ROS, leading to ferroptosis. Together, our study reveals a novel role of DEPDC5 in protecting CD8^+^ T cells from ROS-induced ferroptosis and uncovers a strategy for promoting anti-tumor immunity by suppressing ferroptosis.

## Results

### Epilepsy patients with *DEPDC5* mutation exhibit low blood CD8^+^ T cell counts

**Fig. 1:**
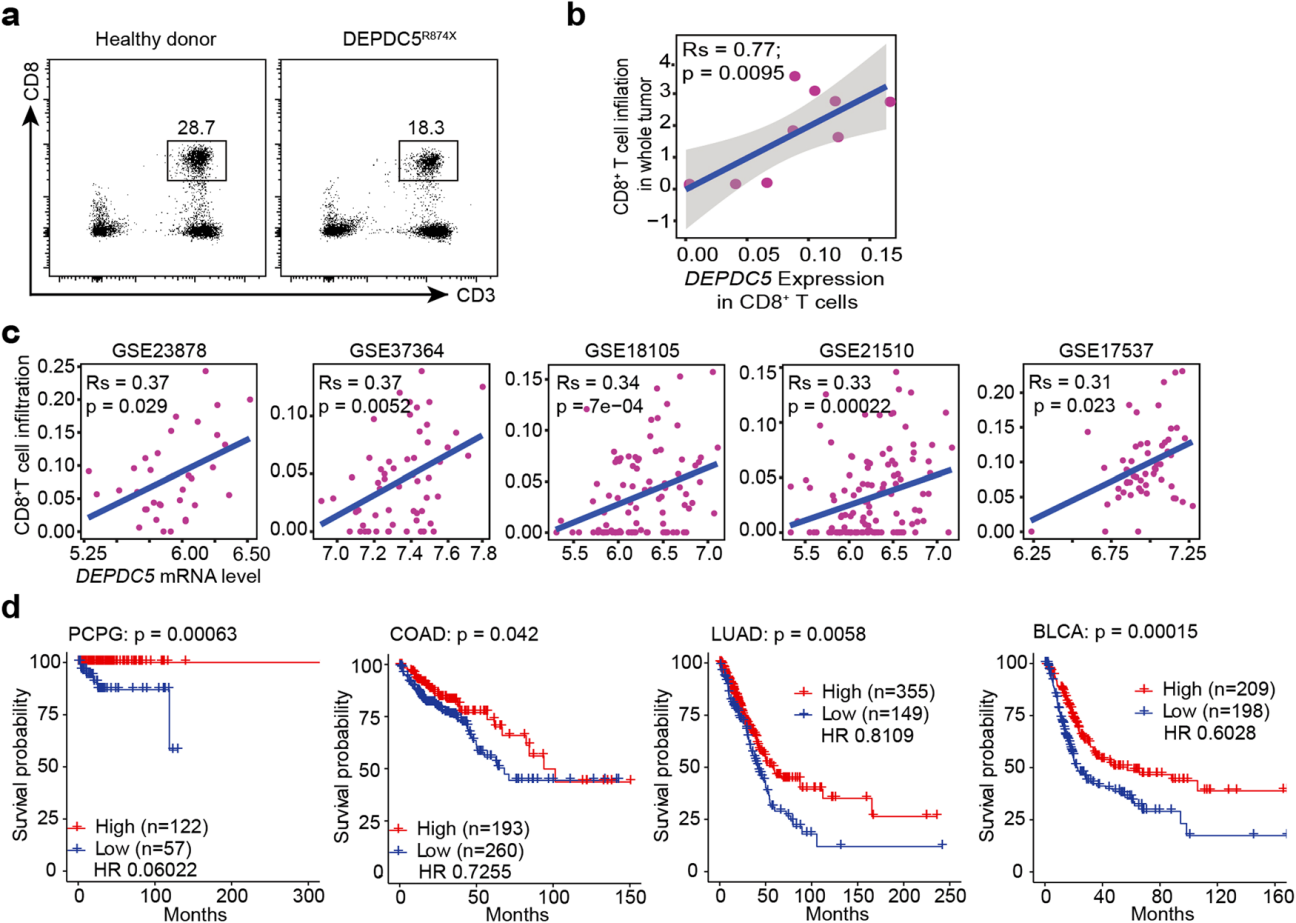
*DEPDC5* expression positively correlates with tumor infiltration by CD8^+^ T cells and overall patient survival. **a** Flow cytometry analysis of peripheral blood mononuclear cells (PBMCs) from healthy donors and patients with *DEPDC5* mutations (*DEPDC5*^*R874X*^), after staining with anti-human CD3 and CD8 antibodies. The number above the box in each panel represents CD3^+^CD8^+^ T cells as a percentage of total PBMCs. **b** Percentage of tumor-infiltrating CD8^+^ T cells correlates with gene expression level of *DEPDC5* within the CD8^+^ population, as analyzed by scRNA-seq of tumor samples from 9 patients with colorectal cancer. **c** Spearman correlation of *DEPDC5* mRNA level with percentage of tumor-infiltrating CD8^+^ T cells across five independent CRC datasets, including GSE23878 (*n* = 35), GSE37364 (*n* = 56), GSE18105 (*n* = 94), GSE21510 (*n* = 123), GSE17537 (*n* = 55). **d** Kaplan-Meier curve indicating overall survival (OS) of patients based on DEPDC5 expression status across 4 types of cancer. PCPG Pheochromocytoma, and paraganglioma, COAD Colonic adenocarcinoma, LUAD Lung adenocarcinoma, BLCA Bladder urothelial carcinoma.

Mutations in the *DEPDC5* gene, which encodes a core component of the GATOR1 complex, have been identified as a major risk factor for epilepsy in children^30^. Through collaboration with physicians at Shanghai Children’s Medical Center, we identified one patient with monoallelic early termination mutation in *DEPDC5* (R847X) who displayed reduced blood CD8^+^ T cell counts compared with a healthy donor (Fig. 1a) and recurrent infection. We also noticed that another epilepsy patient with a frameshift mutation (L1200R frameshift) in *DEPDC5* also had a recurrent infection and reduced blood CD8^+^ T cell numbers compared with healthy donors (data not shown), suggesting that in addition to causing epilepsy, loss of DEPDC5-mediated GATOR1 function may impair CD8^+^ T cell homeostasis.

### CD8^+^ T cell infiltration of human cancer is positively associated with DEPDC5 level

We also determined the DEPDC5 expression and CD8^+^ T cell tumor infiltration in human cancer by analyzing the single-cell RNA sequencing (scRNA-seq) data of tumor samples from nine colorectal cancer (CRC) patients described in an earlier study^31^. We found that the expression level of *DEPDC5* mRNA in CD8^+^ T cells was strongly and positively correlated with the extent of CD8^+^ T cell tumor infiltration (Fig. 1b). In addition, we also observed a strong positive correlation of overall *DEPDC5* expression with the probability of tumor infiltration by CD8^+^ T cells in five independently generated CRC datasets, including GSE23878^32^, GSE37364^33^, GSE18105^34^, GSE21510^35^, and GSE17537^36^ (Fig. 1c), indicating a strong association of *DEPDC5* expression with tumor infiltration by CD8^+^ T cells.

Furthermore, we found that patients with high DEPDC5 expression survived longer than those with low DEPDC5 expression across different tumor types in The Cancer Genome Atlas (TCGA) cohorts, including pheochromocytoma and paraganglioma (PCPG), colonic adenocarcinoma (COAD), lung adenocarcinoma (LUAD), bladder urothelial carcinoma (BLCA) (Fig. 1d). Together, the association of high expression of DEPDC5 with increased tumor-infiltrating CD8^+^ T cells suggests a regulatory role of DEPDC5 in CD8^+^ T cells.

### DEPDC5 is highly expressed in lymphoid tissues

The above results also suggest that DEPDC5 may play a critical role in CD8^+^ T cell immunity. Consistently, data from the Human Protein Atlas (HPA) revealed relatively high expression of *DEPDC5* in immune cells, especially in T lymphocytes^37^. To confirm these data, we used commercial antibodies to examine DEPDC5 protein levels in different mouse tissues/organs, but unfortunately, none of the available antibodies in our hands was effective for detection of endogenous DEPDC5, even when using brain tissue lysate as a positive control (data not shown). For this reason, we instead generated a mouse line on the C57BL/6 background using Turbo Knockout technology^38^ by Cyagen Biosciences (Guangzhou, China) to tag endogenous DEPDC5 with a 3× flag sequence (MDYKDHDGDYKDHDIDYKDDDDK). At the same time, *loxp* sequences were inserted to flank the main ATG codon containing exon 2 of the *Depdc5* gene, thereby facilitating the detection of endogenous DEPDC5 using anti-flag antibodies, but also allowing deletion of this gene in specific tissues in subsequent experiments (illustrated in Supplementary Fig. S1a). For genotype, primers P1 and P2 amplify a 271 bp fragment from the wild-type (WT) allele (+) and a 341 bp fragment from the floxed allele (f) (Supplementary Fig. S1b).

The floxed allele (*Depdc5*^*f*^) encodes a 3× flag-tagged DEPDC5 protein, as confirmed by immunoblotting of brain tissue lysate using an anti-flag antibody (Supplementary Fig. S1c), and was also expressed at high levels in immune organs including thymus, spleen, and lymph nodes (LNs). In contrast, DEPDC5 expression was relatively low in the heart, stomach, small intestine, colon, muscle, liver, kidney, and lung (Supplementary Fig. S1d). Together, these data confirmed the successful generation of a conditional *Depdc5 floxed* mouse line expressing endogenous levels of 3× flag-DEPDC5. Collectively, these findings suggest that DEPDC5 may play important selective roles in both the brain and immune compartment.

### T cell conditional *Depdc5*-deficient mice display reduced CD8^+^ T cell frequencies

Since epilepsy patients with *DEPDC5* mutations exhibit reduced frequencies of CD8^+^ T cells in peripheral blood, we next conditionally knocked out *Depdc5* in T cells using a *Cd4*Cre mouse line^39,40^. The resulting *Cd4*Cre*Depdc5*^*f/f*^ T cell conditional knockout (tko) mice and *Depdc5*^*f/f*^ normal control littermate (ncl) mice are hereafter referred to as *Depdc5*^*tko*^ and *Depdc5*^*ncl*^ mice, respectively. Immunoblotting and reverse transcription quantitative real-time PCR (RT-qPCR) analysis confirmed successful *Depdc5* ablation specifically in CD4^+^ and CD8^+^ T cells but not in B cells, at both protein (Supplementary Fig. S2a) and mRNA (Supplementary Fig. S2b) levels.

**Fig. 2:**
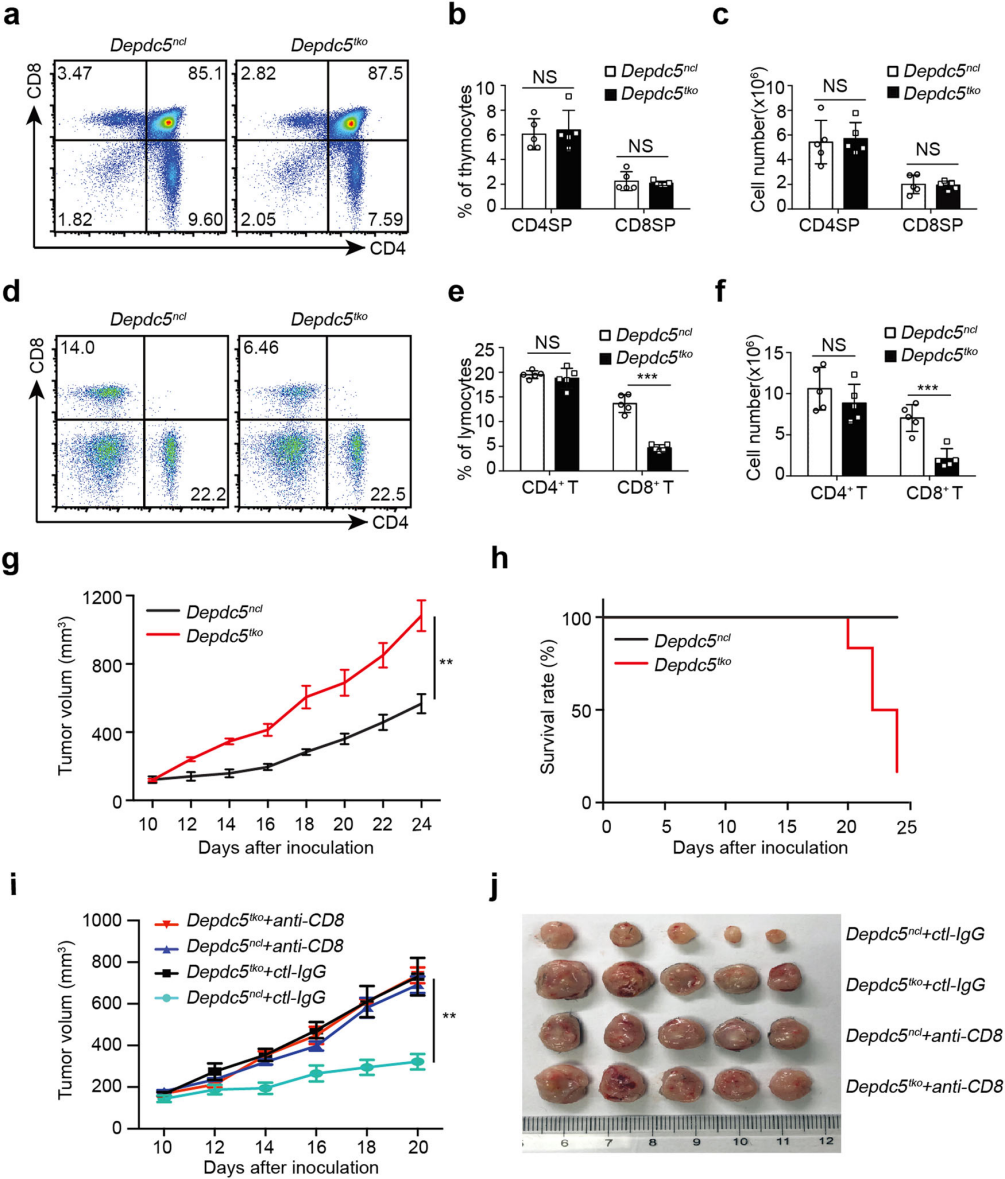
DEPDC5 maintains peripheral CD8^+^ T cell frequency and anti-tumor immunity. **a** Flow cytometry analysis of thymic CD4^+^ and CD8^+^ T cells from *Depdc5*^*ncl*^ and *Depdc5*^*tko*^ mice after staining with anti-mouse CD4 and CD8 antibodies. Numbers in each quadrant show the percentage of the gated populations. **b** Summary bar graph showing the average percentage of CD4SP and CD8SP cells among total thymocytes from *Depdc5*^*ncl*^ and *Depdc5*^*tko*^ mice (*n* = 5). **c** Summary bar graph showing the number of CD4SP and CD8SP thymocytes from *Depdc5*^*ncl*^ and *Depdc5*^*tko*^ mice (*n* = 5). **d** Flow cytometry analysis of splenic CD4^+^ and CD8^+^ T cells from *Depdc5*^*ncl*^ and *Depdc5*^*tko*^ mice after staining with anti-mouse CD4 and CD8 antibodies. Numbers in each quadrant show the percentage of the gated populations. **e** Summary bar graph showing the percentages of splenic CD4^+^ and CD8^+^ T cells in *Depdc5*^*ncl*^ mice and *Depdc5*^*tko*^ mice (*n* = 5). **f** Summary bar graph showing the number of splenic CD4^+^ and CD8^+^ T cells in *Depdc5*^*ncl*^ mice and *Depdc5*^*tko*^ mice (*n* = 5). **g** *Depdc5*^*ncl*^ and *Depdc5*^*tko*^ mice were injected subcutaneously with 5 × 10^5^ MC38 colon cancer cells at day 0. Tumor volumes were measured at the indicated time points after inoculation (*n* = 6). **h** Survival curve of *Depdc5*^*ncl*^ and *Depdc5*^*tko*^ tumor-bearing mice. Animals were sacrificed after tumor volume reached 1000 mm^3^ (then recorded as dead at the corresponding time point). **i** *Depdc5*^*ncl*^ and *Depdc5*^*tko*^ mice were injected subcutaneously with 5 × 10^5^ MC38 colon cancer cells at day 0. Both *Depdc5*^*ncl*^ and *Depdc5*^*tko*^ mice were administered 250 μg/mouse anti-CD8 monoclonal antibody or a control IgG via I.P. injection at day 3, day 6, and day 9 after inoculation. Tumor volumes were measured at the indicated time points after inoculation (*n* = 5). **j** Picture of MC38 tumors from *Depdc5*^*ncl*^ and *Depdc5*^*tko*^ mice that were I.P. injection treated or not with anti-mouse CD8 depletion antibody or control IgG. Mice were sacrificed at day 21 after inoculation and tumors were freshly isolated from subcutaneous tissue. Error bars indicate means ± SEM (NS, not significant, **P < 0.01, ***P < 0.001 by unpaired Student’s *t-*test).

To examine the impact of *Depdc5* deletion on thymic T cells, thymocytes were stained with anti-CD4 and anti-CD8 antibodies for analysis by flow cytometry. As shown in Fig. 2a–c, percentages of CD4 and CD8 double positive (DP), double negative (DN), and CD4^+^CD8^−^ single-positive (CD4SP), or CD4^-^CD8^+^ single-positive (CD8SP) cells were comparable between *Depdc5*^*ncl*^ and *Depdc5*^*tko*^ littermate mice, indicating that *Depdc5* was not required for thymic T cell development or maturation. We also examined the percentages and cell numbers of CD4^+^ and CD8^+^ T cells in the spleen (Fig. 2d–f), mesenteric LN (Supplementary Fig. S2c, d), blood (Supplementary Fig. S2e, f), and bone marrow (Supplementary Fig. S2g, h). We observed that CD8^+^ T cell counts in the spleen, LN, blood, and bone marrow were all significantly reduced in *Depdc5*^*tko*^ mice compared with control littermates; surprisingly, CD4^+^ T cells were minimally affected in those organs of *Depdc5*^*tko*^ mice. Furthermore, we observed a similar deficit in CD8^+^ T cell counts in *Depdc5*^*tko*^ mice at both 6 weeks and 6 months of age (Supplementary Fig. S2i, j), confirming sustained disruption of CD8^+^ T cell homeostasis.

### T cell-specific *Depdc5* deletion impairs anti-tumor immunity

To further investigate the DEPDC5 function in T cells in vivo, MC38 colon carcinoma cells (5 × 10^5^ cells per mouse) were injected subcutaneously into 6-week-old *Depdc5*^*ncl*^ and *Depdc5*^*tko*^ mice, and tumor volumes were measured on alternate days (starting from day 10 after inoculation). As shown in Fig. 2g, tumors grew significantly faster in *Depdc5*^*tko*^ mice than in *Depdc5*^*ncl*^ mice, suggesting a role for DEPDC5 in T cell-mediated anti-tumor immunity. Consistently, *Depdc5*^*tko*^ tumor-bearing mice displayed reduced survival time relative to *Depdc5*^*ncl*^ tumor-bearing mice (Fig. 2h). To determine if impaired anti-tumor immunity was related to CD8^+^ T cell function, *Depdc5*^*ncl*^, and *Depdc5*^*tko*^ tumor-bearing mice were next treated intraperitoneal (I.P.) injection with anti-CD8 depletion antibody (250 μg per mouse per time point) or a control antibody at days 3, 6, and 9 after tumor inoculation. As shown in Fig. 2i, j, administration of anti-CD8 depletion antibody but not control antibody led to comparable tumor growth in both *Depdc5*^*ncl*^ and *Depdc5*^*tko*^ mice, indicating that beneficial anti-tumor responses in *Depdc5*^*ncl*^ mice were mediated by CD8^+^ T cells.

### *Depdc5*-deficient CD8^+^ T cells undergo ferroptosis

**Fig. 3:**
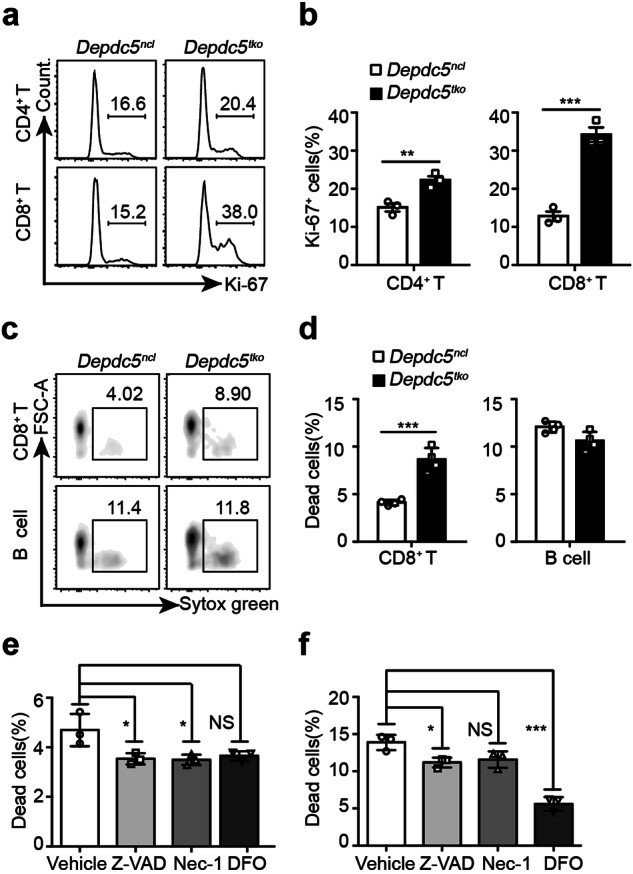
*Depdc5-*deficient CD8^+^ T cells display enhanced ferroptosis. **a** Representative histograms showing Ki-67 levels in splenic CD4^**+**^ and CD8^**+**^ T cells from *Depdc5*^*ncl*^ and *Depdc5*^*tko*^ mice. Splenocytes from *Depdc5*^*ncl*^ and *Depdc5*^*tko*^ mice were stained with a fixable viability dye and antibodies against CD4, CD8, and Ki-67. Numbers in the gated areas show the percentage of Ki-67^+^ cells within the indicated populations. **b** Summary bar graph of percentage Ki-67^+^ cells within the CD4^**+**^ and CD8^**+**^ compartments of *Depdc5*^*ncl*^ and *Depdc5*^*tko*^ mice (*n* = 3). **c** Flow cytometry analysis of dead lymphocytes (Sytox green positive) among splenic CD8^+^ T cells and B cells from *Depdc5*^*ncl*^ and *Depdc5*^*tko*^ mice (after in vitro culture for 4 h with Sytox green viability dye, anti-B220 antibody, and anti-CD8 antibody). **d** Summary bar graphs showing the percentage of dead CD8^+^ T cells and B cells from *Depdc5*^*ncl*^ and *Depdc5*^*tko*^ mice after in vitro culture for 4 h (*n* = 4). **e** Summary bar graph showing the percentage of dead WT CD8^+^ T cells after treatment with vehicle only, pan-caspase inhibitor Z-VAD-FMK (Z-VAD), necroptosis inhibitor Necrostatin-1 (Nec-1), or ferroptosis inhibitor Deferoxamine (DFO). LN cells from *Depdc5*^*ncl*^ mice after in vitro culture for 4 h with vehicle only, Z-VAD, Nec-1, or DFO, then stained with anti-CD8 antibody and fixable viability dye. Total CD8^+^ T lymphocytes were gated for dead cell analysis by flow cytometry. **f** Summary bar graph showing the percentage of dead CD8^+^ T cells after treatment with vehicle only, Z-VAD, Nec-1, or DFO. LN cells from *Depdc5*^*tko*^ mice were cultured in vitro for 4 h in the presence of vehicle only, Z-VAD, Nec-1, or DFO, then stained with anti-CD8 antibody and fixable viability dye for flow cytometry analysis. Total CD8^+^ T cells were gated for dead cell analysis. Error bars indicate means ± SEM (NS, not significant, *P < 0.05, **P < 0.01, ***P < 0.001 by unpaired Student’s *t*-test).

To investigate the mechanism underpinning CD8^+^ T cell defects in *Depdc5*^*tko*^ mice, we next examined lymphocyte proliferation and survival in *Depdc5*^*ncl*^ vs *Depdc5*^*tko*^ animals. As shown in Fig. 3a, b, *Depdc5*-deficient CD4^+^ T cells displayed slightly increased Ki-67 incorporation compared with WT control CD4^+^ T cells, whereas *Depdc5*-deficient CD8^+^ T cells exhibited far higher levels of Ki-67 incorporation than their *Depdc5*^*ncl*^ counterparts. These data suggest that the reduced number of peripheral CD8^+^ T cells in *Depdc5*^*tko*^ mice was more likely related to cell survival than a defect in proliferation. Indeed, we observed significantly greater amounts of cell death among splenic CD8^+^ T cells from *Depdc5*-deficient mice relative to WT, whereas B cell survival was unchanged (Fig. 3c, d), confirming that *Depdc5*-deficient CD8^+^ T cells were more susceptible to cell death.

T cells may undergo cell death via apoptosis, necroptosis, or a more recently discovered pathway termed ferroptosis. To determine which form of cell death *Depdc5*-deficient CD8^+^ T cells might be susceptible to, we treated WT and *Depdc5*-deficient CD8^+^ T cells with Z-VAD-FMK (Z-VAD), Necrostatin-1 (Nec-1), and Deferoxamine (DFO), to suppress apoptosis, necroptosis, or ferroptosis, respectively. As shown in Fig. 3e, 4 h treatment with Z-VAD, Nec-1, and DFO only slightly suppressed cell death in WT CD8^+^ T cells compared with vehicle-treated CD8^+^ T cells. However, the same DFO treatment of *Depdc5*-deficient CD8^+^ T cells led to much stronger suppression of cell death than did treatment with Z-VAD or Nec-1 (Fig. 3f). In addition, we used another ferroptosis inhibitor ferrostatin-1 (Fer-1) in these experiments, which also suppressed *Depdc5*-deficient CD8^+^ T cell death in vitro (Supplementary Fig. S3a, b). These results indicate that *Depdc5* deficiency may augment ferroptosis in CD8^+^ T cells.

To confirm that *Depdc5*-deficient CD8^+^ T cells were more susceptible to cell death by ferroptosis, we next measured lipid ROS levels in WT and *Depdc5-*deficient CD8^+^ T cells using BODIPY™ 581/591 C11^18^. Oxidation of BODIPY™ 581/591 C11’s polyunsaturated butadienyl portion shifts the fluorescence emission peak from ~590 nm to ~510 nm, which can be measured by flow cytometry^41^. As shown in Supplementary Fig. S3c, d, *Depdc5*-deficient CD8^+^ T cells displayed much higher levels of lipid ROS than did their WT counterparts. Although lipid ROS was also increased in *Depdc5*-deficient CD4^+^ T cells compared to WT CD4^+^ T cells, this increased lipid ROS level in *Depdc5*-deficient CD4^+^ T cells remained significantly lower than the levels observed in *Depdc5*-deficient CD8^+^ T cells (Supplementary Fig. S3d). Importantly, both ferroptosis inhibitors Fer-1 and DFO could suppress the increased lipid ROS levels in *Depdc5*-deficient CD8^+^ T cells (Supplementary Fig. S3e, f). These data suggest that DEPDC5 may protect CD8^+^ T cells from ferroptosis by reducing lipid ROS levels.

Cellular ROS produced by the reaction of H_2_O_2_ and Fe^2+^ is required for the oxidation of PUFA-containing phospholipids (PUFA-PLs) and subsequent induction of ferroptosis^20^, so we next used the cell-permeable probe H_2_DCFDA to measure ROS levels in WT and *Depdc5-*deficient CD4^+^ and CD8^+^ T cells (Supplementary Fig. S3g, h). Again, we observed that *Depdc5*-deficient CD8^+^ T cells exhibited far higher ROS levels than did WT CD8^*+*^ T cells. Intriguingly, *Depdc5*-deficient CD4^+^ T cells also displayed higher ROS levels than WT CD4^+^ T cells, indicating that additional factors may be involved in the ferroptosis of *Depdc5*-deficient CD8^+^ T cells in vivo.

### Vitamin E supplementation or iron-free diet rescue *Depdc5*^*tko*^ CD8^+^ T cells

**Fig. 4:**
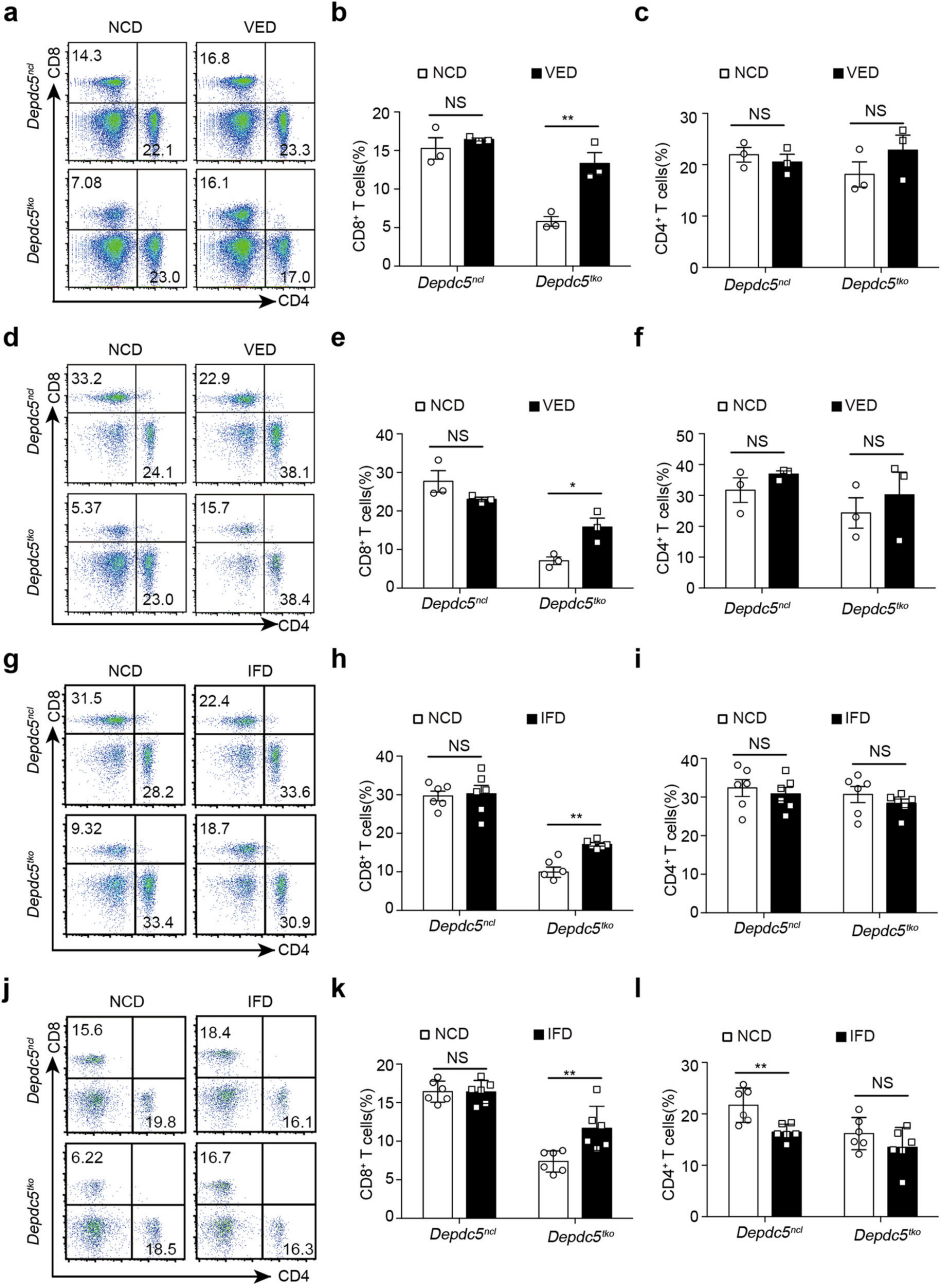
Ferroptosis suppression rescues CD8^+^ T cells in *Depdc5*^*tko*^ mice. **a** Flow cytometry analysis of splenic CD4^+^ and CD8^+^ T cells from *Depdc5*^*ncl*^ and *Depdc5*^*tko*^ mice fed with NCD or VED for 4 weeks. Numbers in quadrants show the percentage of the gated subsets. **b**, **c** Summary bar graphs showing the percentage of splenic CD8^+^ T cells (**b**) and CD4^+^ T cells (**c**) from *Depdc5*^*ncl*^ and *Depdc5*^*tko*^ mice as described in **a** (*n* = 3). **d**–**f** Representative flow cytometry plots (**d**), and bar graphs showing the percentage of CD8^+^ T cells (**e**) and CD4^+^ T cells (**f**) in LN from *Depdc5*^*ncl*^ and *Depdc5*^*tko*^ mice fed with NCD or VED for 4 weeks as in **a** (*n* = 3). **g** Flow cytometry analysis of LN CD4^+^ and CD8^+^ T cells from *Depdc5*^*ncl*^ and *Depdc5*^*tko*^ mice fed with NCD or IFD for 4 weeks. Numbers in quadrants show the percentage of the gated subsets. **h**, **i** Summary bar graphs show the percentage of CD8^+^ T cells (**h**) and CD4^+^ T cells (**i**) from *Depdc5*^*ncl*^ and *Depdc5*^*tko*^ mice as described in **g** (*n* = 6). **j**–**l** Representative flow cytometry plots (**j**), and bar graphs showing the percentage of CD8^+^ T cells (**k**) and CD4^+^ T cells (**l**) in blood from *Depdc5*^*ncl*^ and *Depdc5*^*tko*^ mice fed with NCD or IFD for 4 weeks (*n* = 6). Error bars indicate means ± SEM. (NS, not significant, *P < 0.05, **P < 0.01 by unpaired Student’s *t*-test).

We next assessed whether ferroptosis was responsible for reduced peripheral CD8^+^ T cell counts in *Depdc5*^*tko*^ mice in vivo. To investigate this, we fed *Depdc5*^*ncl*^ and *Depdc5*^*tko*^ mice daily with a normal chow diet (NCD) or supplemented with 1000 mg/kg Vitamin E (VED), since Vitamin E has been shown to suppress ferroptosis by inhibiting ROS production both in vitro and in vivo^22,23^. As predicted, feeding *Depdc5*^*tko*^ mice with VED led to a significant increase in CD8^+^ T cell frequency in the spleen (Fig. 4a, b), LN (Fig. 4d, e), and blood (Supplementary Fig. S4a, b) compared with mice fed NCD alone. In contrast, there was minimal difference between the peripheral CD8^+^ T cell compartments of *Depdc5*^*ncl*^ mice fed with VED or NCD (Fig. 4a, b, d, e; Supplementary S4a, b). Furthermore, VED feeding had little impact on peripheral CD4^+^ T cell frequencies in either *Depdc5*^*ncl*^ or *Depdc5*^*tko*^ mice (Fig. 4a, c, d, f), indicating that CD4^+^ and CD8^+^ T cells were differentially regulated by DEPDC5. These results strongly suggest that the loss of peripheral CD8^+^ T cells in *Depdc5*^*tko*^ mice is due to ROS-mediated ferroptosis.

To further determine if *Depdc5*-deficient CD8^+^ T cells die from iron-dependent, ROS-mediated ferroptosis, we next fed *Depdc5*^*ncl*^ and *Depdc5*^*tko*^ littermate mice with iron-free diet (IFD) for 4 weeks. It was reported previously that mice receiving an iron-deficient diet become lymphopenic^42^, and we also observed a small but consistent decrease in total LN lymphocytes in both *Depdc5*^*ncl*^ and *Depdc5*^*tko*^ mice receiving IFD. While in *Depdc5*^*ncl*^ mice the percentage of CD8^+^ T cells remained unchanged, we observed a significant increase in this compartment in *Depdc5*^*tko*^ mice receiving IFD (Fig. 4g, h). Intriguingly, CD4^+^ T cell frequency was similar in *Depdc5*^*ncl*^ or *Depdc5*^*tko*^ mice irrespective of whether these animals were being fed with NCD or IFD (Fig. 4g, i). Blood CD8^+^ T cell counts in *Depdc5*^*tko*^ mice were also increased in the IFD group relative to the NCD group (Fig. 4j, k), whereas blood CD4^+^ T cell frequencies were slightly decreased in *Depdc5*^*ncl*^ mice receiving IFD compared with NCD (Fig. 4j, l). These data demonstrate that while an iron-deficient diet may globally impact both CD4^+^ and CD8^+^ T cell populations, this intervention selectively rescued *Depdc5*-deficient CD8^+^ T cells by suppressing ferroptosis.

### Hyper-mTORC1 activity drives CD8^+^ T cell ferroptosis in *Depdc5*^*tko*^ mice

Since DEPDC5 is a negative regulator of mTORC1, we next examined the activity of this pathway in WT and *Depdc5*-deficient CD8^+^ T cells by measuring phosphorylation of downstream factors S6K, S6, and 4E-BP1 (S6K-pT389, S6-pS235/236 and 4E-BP1-pT37/46). As expected, CD4^+^ and CD8^+^ T cells exhibited significantly higher levels of S6 S235/236 and 4E-BP1 T37/46 phosphorylation in *Depdc5*^*tko*^ relative to *Depdc5*^*ncl*^ mice, whereas non-T (CD3 negative) cells displayed similar phosphorylation patterns in both genotypes (Supplementary Fig. S5a, b). In accordance with higher mTORC1 activity, *Depdc5*-deficient CD8^+^ T cells were also bigger in size than WT CD8^+^ T cells (Supplementary Fig. S5c). Those results suggest that augmented mTORC1 activity may trigger CD8^+^ T cell ferroptosis in *Depdc5*^*tko*^ mice.

**Fig. 5:**
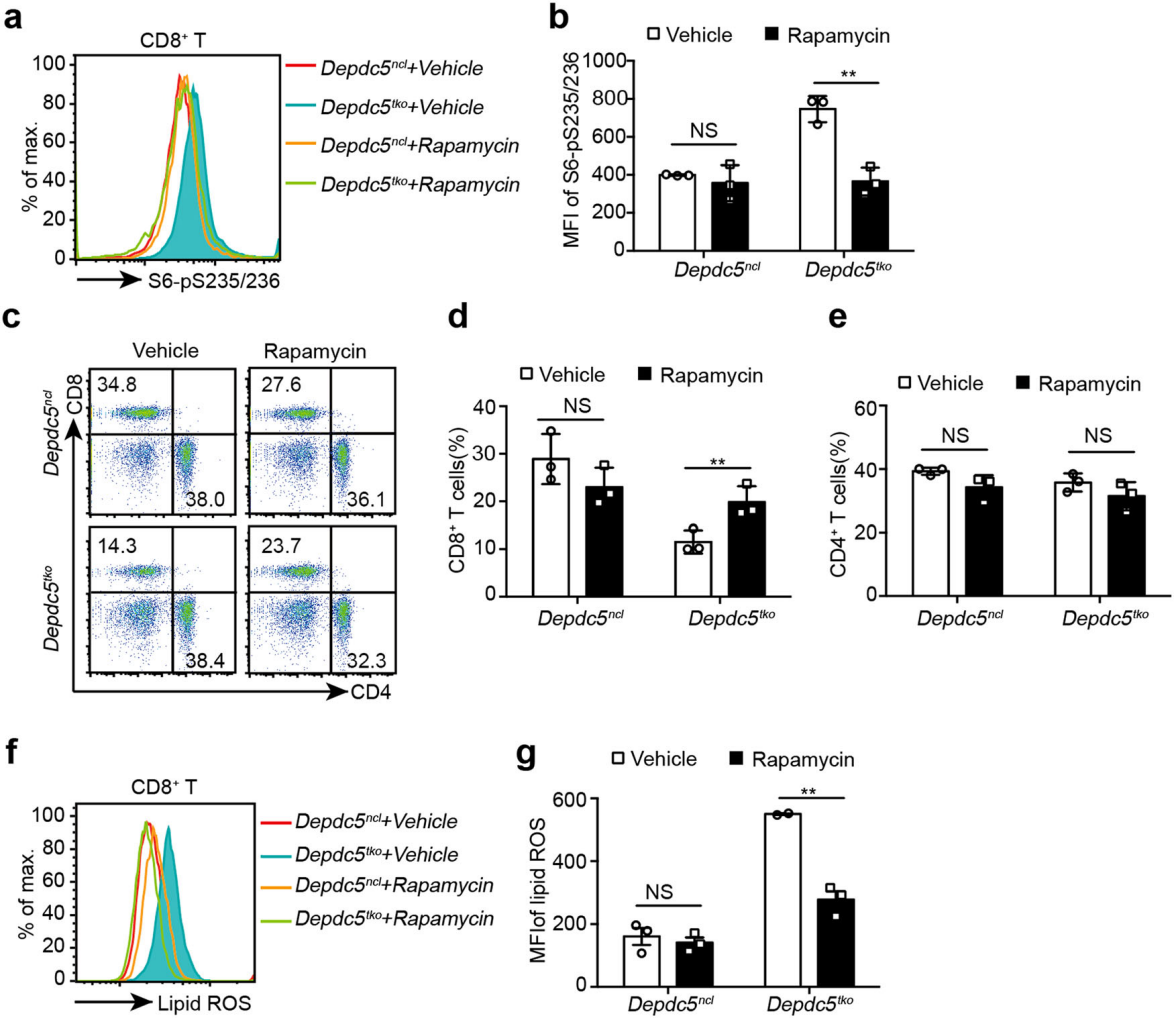
Rapamycin treatment increases blood CD8^+^ T cell frequency in *Depdc5*^tko^ mice. **a** Representative histograms showing phosphorylated S6-S235/236 levels in splenic CD8^+^ T cells from *Depdc5*^*ncl*^ and *Depdc5*^*tko*^ mice after treatment with vehicle only or rapamycin (100 μg/kg daily for 4 weeks). After treatment, splenocytes were isolated and stained with a fixable viability dye, anti-mouse CD8 antibody, and anti-S6-S235/236 phosphorylation antibody for analysis by flow cytometry. **b** Summary bar graph showing MFI of phosphorylated S6-S235/236 in splenic CD8^+^ T cells from *Depdc5*^*ncl*^ and *Depdc5*^*tko*^ mice (*n* = 3) as described as **a**. **c**–**e** Representative flow cytometry plots (**c**) and summary bar graphs showing percentage of CD8^+^ T cells (**d**) and CD4^+^ T cells (**e**) in LN from *Depdc5*^*ncl*^ and *Depdc5*^*tko*^ mice treated with rapamycin or vehicle only as in **a**. LN single cell suspensions were prepared from *Depdc5*^*ncl*^ and *Depdc5*^*tko*^ mice treated daily with vehicle only or 100 μg/kg rapamycin for 4 weeks prior to staining with a fixable viability dye and antibodies against CD4 and CD8 prior to flow cytometry analysis. **f, g** Representative histogram (**f**) and a summary bar graph (**g**) showing relative lipid ROS levels in splenic CD8^+^ T cells from *Depdc5*^*ncl*^ and *Depdc5*^*tko*^ mice treated with vehicle only or rapamycin for 4 weeks. Splenocytes from the same mice in **a** were cultured in the presence of 2 μM BODIPY™ 581/591 C11 for 4 h, washed twice in PBS, then stained with a fixable viability dye and antibodies against CD4 and CD8 for flow cytometry analysis. Error bars indicate means ± SEM. (NS, not significant, **P < 0.01 by unpaired Student’s *t*-test).

To further examine this possibility, *Depdc5*^*ncl*^, and *Depdc5*^*tko*^ littermate mice were treated daily with vehicle or 100 μg/kg rapamycin for 4 weeks before assessing splenic CD8^+^ T cells for mTORC1 activity. WT CD8^+^ T cells exhibited similar S6 and S6K phosphorylation profiles in both vehicle- and rapamycin-treated *Depdc5*^*ncl*^ littermate mice (Fig. 5a, b; Supplementary Fig. S5d, e). In contrast, S6 and S6K phosphorylation was significantly reduced in *Depdc5*-deficient CD8^+^ T cells upon rapamycin treatment, almost decreasing to the level observed in WT CD8^+^ T cells (Fig. 5a, b; Supplementary S5d, e). At the cellular level, rapamycin treatment led to a slight reduction in the percentage of LN CD8^+^ T cells isolated from *Depdc5*^*ncl*^ mice compared to vehicle-treated *Depdc5*^*ncl*^ mice (Fig. 5c, d). In contrast, the same rapamycin treatment led to a marked increase in LN CD8^+^ T cells in *Depdc5*^*tko*^ mice relative to vehicle-treated *Depdc5*^*tko*^ mice (Fig. 5c, d), almost reaching the level of CD8^+^ T cells recovered from rapamycin-treated *Depdc5*^*ncl*^ animals. Intriguingly, CD4^+^ T cells were not significantly impacted upon rapamycin treatment of either *Depdc5*^*ncl*^ or *Depdc5*^*tko*^ mice (Fig. 5c, e), indicating differential roles for DEPDC5 in the CD4^+^ and CD8^+^ compartments. Similar results were obtained for CD4^+^ and CD8^+^ T cells isolated from the spleen (Supplementary Fig. S5f–h), and peripheral blood (Supplementary Fig. S5i–k) of vehicle- and rapamycin-treated *Depdc5*^*ncl*^ and *Depdc5*^*tko*^ mice. Together, these results strongly suggest that reduced CD8^+^ T cell frequencies in *Depdc5*^*tko*^ mice are due to hyper-mTORC1 activity following *Depdc5* deletion.

Given that *Depdc5*-deficient CD8^+^ T cells displayed far higher levels of lipid ROS (Supplementary Fig. S3c, d), we also examined levels of these compounds in splenic CD8^+^ T cells in both *Depdc5*^*ncl*^ and *Depdc5*^*tko*^ mice following rapamycin treatment. In WT CD8^+^ T cells, lipid ROS levels were comparable between vehicle- and rapamycin-treated mice (Fig. 5f, g). In contrast, rapamycin treatment effectively suppressed lipid ROS in *Depdc5*-deficient CD8^+^ T cells, which exhibited levels comparable with WT CD8^+^ T cells (Fig. 5f, g). Since feeding with VED could rescue *Depdc5*-deficient CD8^+^ T cells in *Depdc5*^*tko*^ mice (Fig. 4a, b, d, e; Supplementary S4a, b), we also examined if it may affect mTORC1 activity by measuring S6 phosphorylation. As shown in Supplementary Fig. S4c, no difference in S6 phosphorylation was observed in *Depdc5*-deficient CD8^+^ T cells from either NCD- and VED-fed mice, indicating that lipid ROS and ferroptosis were downstream of mTORC1. Together, these results demonstrate that hyper-mTORC1 activity in *Depdc5*-deficient CD8^+^ T cells augments lipid peroxidation and ferroptosis.

### DEPDC5 restricts the expression of genes encoding purine metabolic enzymes

**Fig. 6:**
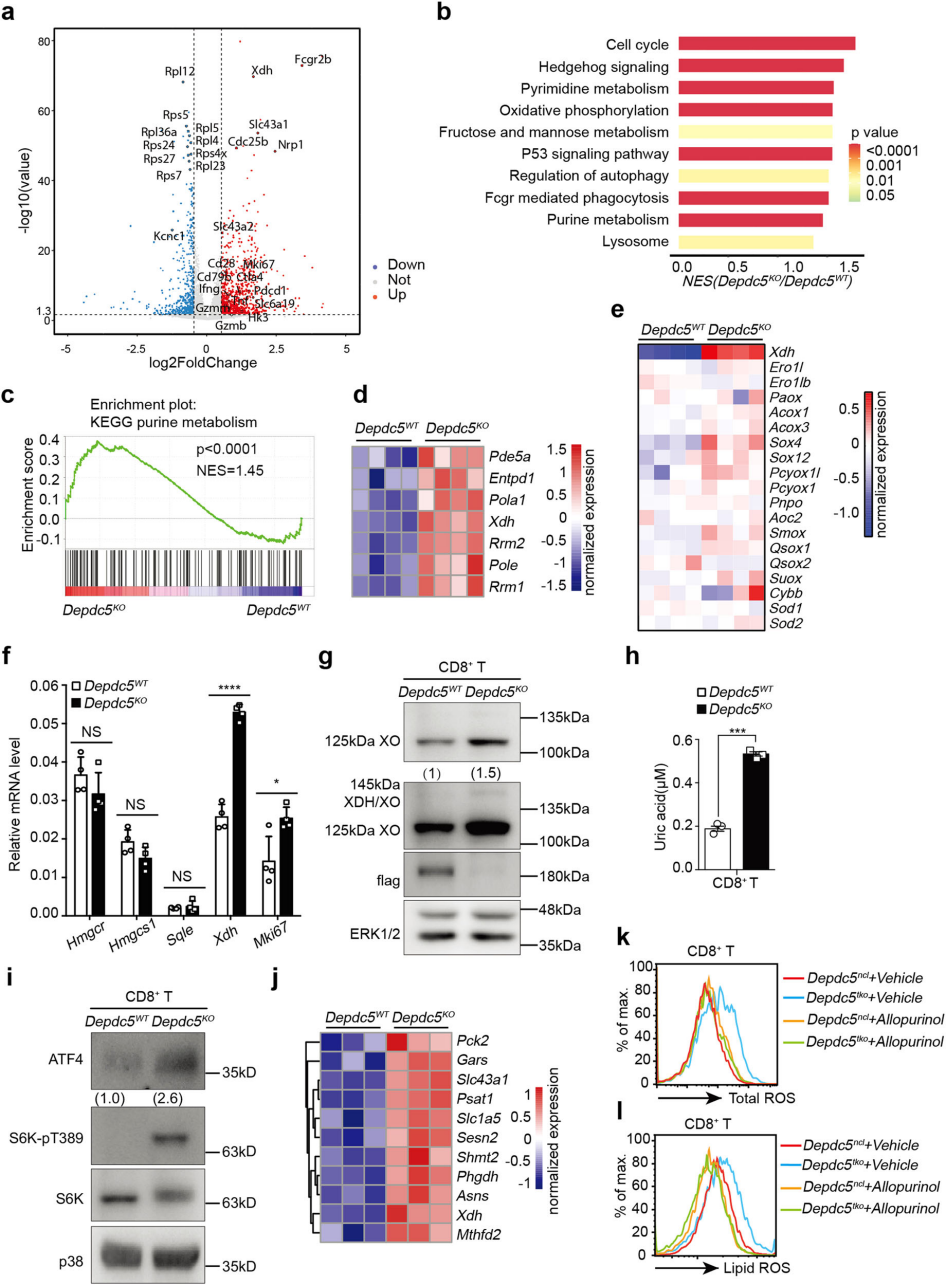
*Depdc5-*deficient CD8^+^ T cells display enhanced expression of purine catabolic enzyme XO due to hyper-mTORC1-ATF4 activation. **a** Volcano plot of differentially expressed genes (DEGs) generated by RNA-seq analysis of *Depdc5* WT (*Depdc5*^*WT*^) and *Depdc5* knock-out (*Depdc5*^*KO*^) CD8^+^ T cells. Red dots represent genes significantly upregulated in *Depdc5*^*KO*^ CD8^+^ T cells (*P* < 0.05 and log_2_ fold change (FC )≥ 0.5), while blue dots represent genes significantly downregulated (*P* < 0.05 and log_2_ FC ≤ 0.5), and grey dots indicate DEGs below the level of significance. **b** KEGG pathway analysis of upregulated DEGs in **a**. The top 10 significant KEGG pathways based on upregulated genes are presented by normalized enrichment score (NES) and *P* value. **c** Gene set enrichment of “KEGG purine metabolism” pathway in *Depdc5*^*KO*^ relative to *Depdc5*^*WT*^ CD8^+^ T cells. **d** Heatmap showing normalized expression of purine metabolism-linked genes that were significantly up-regulated in *Depdc5*^*KO*^ CD8^+^ T cells (*n* = 4). **e** Heatmap showing normalized expression of genes encoding ROS-generating enzymes in *Depdc5*^*WT*^ and *Depdc5*^*KO*^ CD8^+^ T cells (*n* = 4). **f** RT-qPCR analysis of *Xdh*, *Mki67*, and related anti-ferroptotic mevalonate pathway genes *Hmgcr*, *Hmgcs1*, and *Sqle* expression in CD8^+^ T cells from *Depdc5*^*ncl*^ and *Depdc5*^*tko*^ mice. Relative mRNA levels were normalized to *Gapdh* mRNA level (*n* = 4). **g** Immunoblotting of XO protein level in *Depdc5*^*WT*^ and *Depdc5*^*KO*^ splenic CD8^+^ T cells. XO molecular weight is 145 kDa (long form) or 125 kDa (short form) while XDH molecular weight is 145 kDa. ERK1/2 served as a loading control. Numbers under the XO immunoblotting bands indicate the density of XO relative to ERK1/2. **h** Bar graph showing analysis of uric acid levels in *Depdc5*^*WT*^ and *Depdc5*^*KO*^ CD8^+^ T cells (*n* = 3). **i** Immunoblotting of ATF4 protein level in *Depdc5*^*WT*^ and *Depdc5*^*KO*^ splenic CD8^+^ T cells. S6K and S6K-pT389 profiles were assessed to determine mTORC1 activity, while p38 served as a loading control. **j** Heatmap showing normalized expression of ATF4 target genes in *Depdc5*^*WT*^ and *Depdc5*^*KO*^ CD8^+^ T cells (*n* = 3). **k** Cellular ROS levels in *Depdc5*^*WT*^ and *Depdc5*^*KO*^ splenic CD8^+^ T cells treated for 4 h in vitro with either vehicle alone or XO inhibitor allopurinol. **l** Relative lipid peroxidation levels in *Depdc5*^*WT*^ and *Depdc5*^*KO*^ splenic CD8^+^ T cells treated as in **k**. Error bars indicate means ± SEM (NS, not significant, *P < 0.05, ***P < 0.001, ****P < 0.0001 by unpaired Student’s *t*-test).

To search for key genes downstream of DEPDC5, we performed RNA sequencing (RNA-seq) of splenic CD8^+^ T cells from *Depdc5*^*ncl*^ and *Depdc5*^*tko*^ mice. When compared with WT CD8^+^ T cells, *Depdc5-*deficient CD8^+^ T cells displayed upregulation of 867 genes and downregulation of 570 genes (Fig. 6a). Pathway enrichment analysis revealed that *Depdc5-*deficient CD8^+^ T cells display enhanced expression of genes related to cell cycle regulation^43^ and oxidative phosphorylation^44^ (Fig. 6b), consistent with their increased proliferative capacity (Fig. 3a, b). Using gene ontology (GO) enrichment analysis, we also observed that *Depdc5-*deficient CD8^+^ T cells exhibit reduced expression of genes related to rRNA metabolic processes and biogenesis of ribosomal small and large subunits, in parallel with increased expression of genes related to T cell activation, cytokine production, and proliferation (Supplementary Fig. S6a). These results suggest that DEPDC5 may be required for ribosomal biogenesis while also limiting T cell activation and proliferation. Intriguingly, *Depdc5-*deficient CD8^+^ T cells were also observed to display increased expression of genes associated with metabolism of pyrimidine^45^ and purine^46^ (Fig. 6a–c), including *Xdh, Pde5a, Entpd1, Pola1, Rrm2, Pole, and Rrm1* (Fig. 6d). Having already established that *Depdc5-*deficient CD8^+^ T cells exhibit increased mTORC1 activity, which plays a key role in anabolic metabolism, these findings strongly suggested that DEPDC5 may specifically regulate the mTORC1-mediated pyrimidine/purine metabolic pathway.

### DEPDC5 does not directly regulate the expression of genes known to induce ferroptosis

Since ferroptosis induction requires both iron and cellular ROS^47,48^, we next examined genes that encode enzymes associated with ROS production, including *Xdh*, *Ero1l*, *Ero1lb*, *Paox, Acox1*, *Acox3*, *Sox4*, *Sox12*, *Pcyox1l*, *Pcyox1*, *Pnpo*, *Aoc2*, *Smox*, *Qsox1*, *Qsox2*, *Suox*, *Cybb*, *Sod1* and *Sod2*^49^. Surprisingly, *Xdh* was found to be dramatically increased in *Depdc5*-deficient relative to WT CD8^+^ T cells, whereas other genes were either not detected or expressed at comparable levels in both genotypes (Fig. 6e). Augmented expression of *Xdh* in *Depdc5*-deficient CD8^+^ T cells was also confirmed via RT-qPCR analysis (Fig. 6f), consistent with the above mentioned finding that *Xdh* is the main gene associated with purine metabolism upregulated in these cells (Fig. 6d). Together, these data strongly indicate a link between elevated purine metabolism, upregulation of ROS, and susceptibility to ferroptosis in *Depdc5*-deficient CD8^+^ T cells.

We also examined the relative mRNA levels of known genes associated with ferroptosis inhibition e.g. *Gpx4, Fsp1(Aifm2), Slc3a2, Gclc*, and *Fancd2*, or ferroptosis induction/augmentation e.g. *Atp5g3, Acsf2, Vdac2*, *Acsl4, Sat1, Lpcat3, Tfrc, and Ncoa4*, in WT and *Depdc5*-deficient CD8^+^ T cells (Supplementary Fig. S6b)^16,18,50–54^. We found that all ferroptosis-related genes that we checked were expressed at similar levels in both genotypes, except for *Fancd2* (Supplementary Fig. S6b). Since FANCD2 has been identified as a ferroptosis suppressor^54^, up-regulation of this gene in *Depdc5*-deficient CD8^+^ T cells seemed unlikely to explain the enhanced ferroptosis observed in these cells. Together, these results suggest that DEPDC5 defects augment purine metabolism by promoting hyper-mTORC1 activity, thereby increasing expression of *Xdh* whose product is the ROS-producing enzyme xanthine oxidoreductase (XOR), which in turn enhances CD8^+^ T cell ferroptosis via augmented ROS production.

### Augmented *Xdh* expression in *Depdc5*-deficient CD8^+^ T cells results in elevated levels of short-form XO and ROS production

We next examined protein levels of the XOR enzyme encoded by *Xdh* (the only purine metabolism gene found to be differentially expressed and was likely to influence ROS production in *Depdc5*-deficient CD8^+^ T cells). XOR can exist as both a long-form 145 kDa protein and short-form 125 kDa protein. The long form can function either as a Xanthine Dehydrogenase (XDH) that uses NAD^+^ to produce NADH or alternatively, as an XO that employs O_2_ to produce H_2_O_2_, respectively to catalyze the same hypoxanthine to xanthine and xanthine to uric acid reactions^55^. Intriguingly, the short-form 125 kDa protein is generated from the long form via irreversible proteolytic processing, resulting in an enzyme that is only capable of XO function and mainly produces H_2_O_2_ (ROS)^55^.

While both long and short forms of XOR were found to be expressed in non-immune organs (lung and liver), 125 kDa XO was preferentially expressed in the spleen and thymus (Supplementary Fig. S6c). Consistently, long-form XOR was expressed at a low level whereas short-form XO was the major *Xdh* gene product detected in both WT and *Depdc5*-deficient CD8^+^ T cells (Fig. 6g). Importantly, expression levels of short-form XO were dramatically increased in *Depdc5*-deficient CD8^+^ T cells relative to WT CD8^+^ T cells (Fig. 6g). Furthermore, we observed that the uric acid end product of XO-mediated purine metabolism^56,57^, was also significantly increased in *Depdc5*-deficient CD8^+^ T cells compared with WT CD8^+^ T cells (Fig. 6h). These data further indicated that the 125 kDa short-form XO is selectively upregulated in *Depdc5*-deficient CD8^+^ T cells.

### *Xdh* is a major target gene downstream of mTORC1 in CD8^+^ T cells

If *Xdh* was the main target suppressed by DEPDC5 in CD8^+^ T cells, we reasoned that this gene was likely also a downstream target of mTORC1. Indeed, it was previously reported mTORC1-mediated control of transcription factor ATF4^46,58^, plays a key role in regulating *Xdh* to promote purine metabolism in the presence of high glucose^59^. Given our observation that both mTORC1 activity and purine metabolism are upregulated in *Depdc5*-deficient CD8^+^ T cells, we postulated that ATF4 expression may also be increased. Indeed, ATF4 protein level was found to be much higher in *Depdc5*-deficient CD8^+^ T cells than that in WT CD8^+^ T cells (Fig. 6i). In line with this finding, many known ATF4 target genes were also upregulated in *Depdc5*-deficient CD8^+^ T cells relative to WT (including *Pck2*, *Gars*, *Slc43a1*, *Psat1*, *Slc1a5*, *Sesn2*, *Shmt2*, *Phgdh*, *Asns*, *Xdh*, and *Mthfd2*)^58,60^ (Fig. 6j). In addition, mTORC1 inhibition effectively reduced ATF4 protein levels in HEK-293 and HT-1080 cells (Supplementary Fig. S6d), whereas ATF4 deletion decreased XO expression in Cal-1 cells (Supplementary Fig. S6e). Together, these results reveal that DEPDC5-regulated mTORC1 activity controls XO enzyme levels in CD8^+^ T cells via effects on transcription factor ATF4.

### XO drives ROS production and lipid peroxidation in *Depdc5*-deficient CD8^+^ T cells

To confirm that the increased XO activity in *Depdc5*-deficient CD8^+^ T cells was responsible for elevated ROS production and lipid peroxidation, we treated WT and *Depdc5*-deficient CD8^+^ T cells either with vehicle or well-known XOR inhibitor allopurinol (which is in frequent clinical use for the treatment of gout, a metabolic disease linked with enhanced purine metabolism)^57^. While allopurinol exerted minimal effect on WT CD8^+^ T cells, this same treatment led to significant inhibition of total intracellular ROS (Fig. 6k) and lipid ROS levels (Fig. 6l) in *Depdc5*-deficient CD8^+^ T cells. To test whether allopurinol could also rescue CD8^+^ T cells in *Depdc5*^*tko*^ mice in vivo, *Depdc5*^*ncl*^ and *Depdc5*^*tko*^ mice were treated with 10 mg/kg allopurinol daily by I.P. injection for 4 weeks before their splenic CD8^+^ T cells were analyzed. We found that allopurinol treatment significantly increased the percentages of CD8^+^ T cells in *Depdc5*^*tko*^ mice as compared to that in the vehicle-treated group, but had little effect on CD8^+^ T cell population in *Depdc5*^*ncl*^ mice in vivo (Supplementary Fig. S6f, g). These data directly linked increased XO expression and activity with the impairment of CD8^+^ T cells in *Depdc5*^*tko*^ mice.

### Inhibition of ferroptosis restores anti-tumor immunity in *Depdc5*^*tko*^ mice

**Fig. 7:**
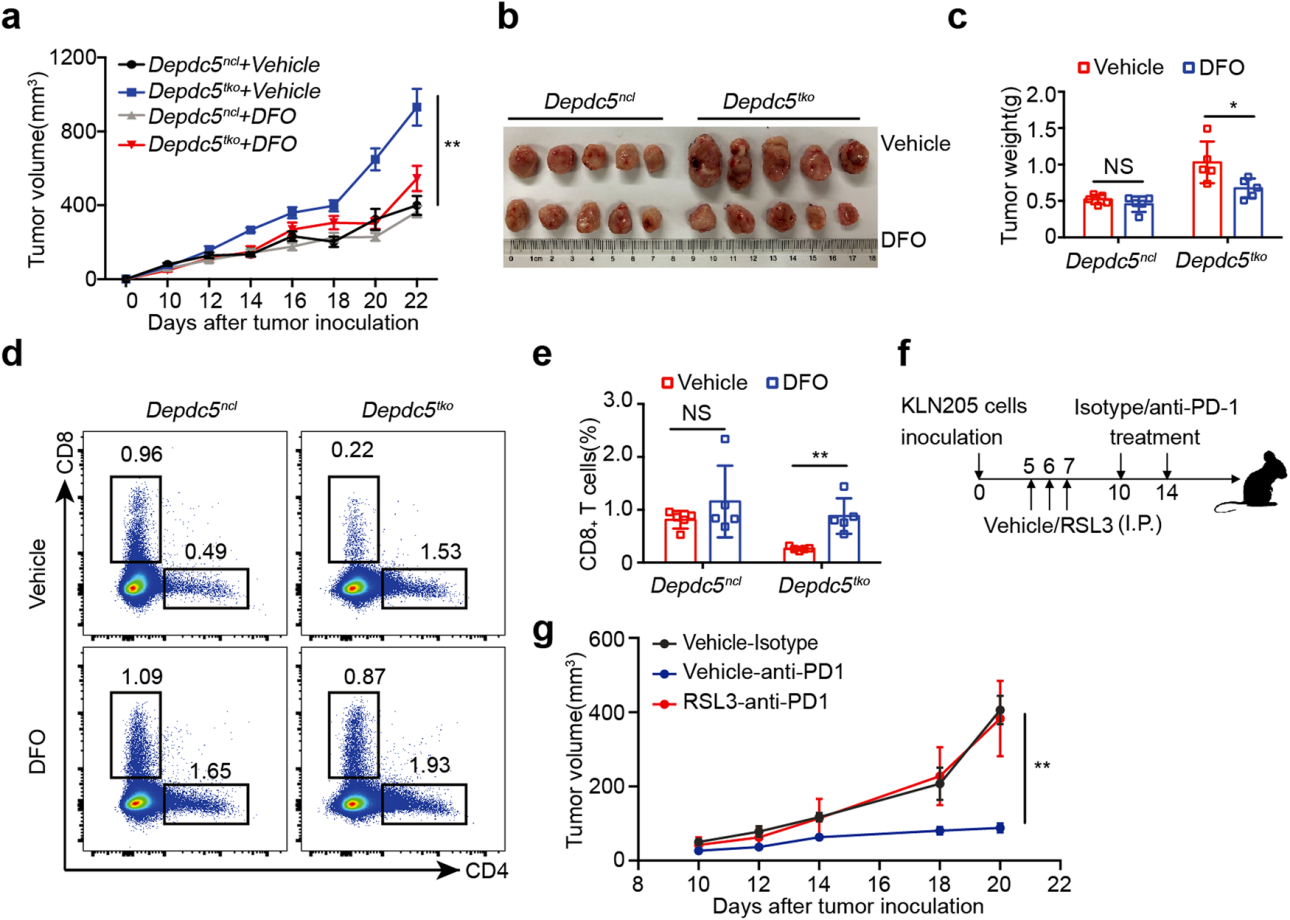
Ferroptosis inhibitor DFO restores anti-tumor immunity in *Depdc5*^*tko*^ mice. **a** *Depdc5*^*ncl*^ and *Depdc5*^*tko*^ mice were injected subcutaneously with 5 × 10^5^ MC38 tumor cells at day 0. Both *Depdc5*^*ncl*^ and *Depdc5*^*tko*^ mice were divided into two groups: the first group received I.P. injection with 200 μg/kg/day DFO, while the second group received I.P. injection vehicle only, beginning day 1 after tumor inoculation. Tumor volumes were then measured at the indicated time points (*n* = 5). **b** Photograph of MC38 tumors from *Depdc5*^*ncl*^ and *Depdc5*^*tko*^ mice treated with vehicle only or DFO prior to sacrifice on day 23 after tumor inoculation (*n* = 5). **c** Summary bar graph showing tumor weight in *Depdc5*^*ncl*^ and *Depdc5*^*tko*^ mice treated with vehicle only or DFO prior to sacrifice on day 23 after tumor inoculation. **d** Flow cytometry analysis of tumor-infiltrating CD4^+^ and CD8^+^ T cell percentages within the CD45^+^ pool of MC38 tumors from *Depdc5*^*ncl*^ and *Depdc5*^*tko*^ mice treated with vehicle or DFO. At day 23 after tumor inoculation, single-cell suspensions of each tumor were isolated from individual mice, then stained with a fixable viability dye and antibodies against CD45 and CD8 prior to analysis by flow cytometry. **e** Summary bar graph showing tumor-infiltrating CD8^+^ T cell percentage within the CD45^+^ pool of tumor tissue as in **d**. **f** Mice were inoculated with KLN205 cells, treated with vehicle or RSL3 from day 5 to day 7, and received anti-PD1 or isotype control antibodies on day 10 and day 14. **g** Comparison of KLN205 tumor growth curve in Vehicle-Isotype, Vehicle-anti-PD1, and RSL3-anti-PD1 conditions treated as **f**. Error bars indicate means ± SEM (NS, not significant, *P < 0.05, **P < 0.01 by unpaired Student’s *t*-test).

We next tested whether inhibition of ferroptosis could restore CD8^+^ T cell survival and anti-tumor function in *Depdc5*^*tko*^ mice. Specifically, *Depdc5*^*ncl*^ and *Depdc5*^*tko*^ littermate mice were pre-inoculated with MC38 colon carcinoma cells and then treated daily with either vehicle or iron-chelating agent DFO. Tumor growth was measured at day 10 after inoculation. As shown in Fig. 7a–c, DFO treatment exerted a minimal effect on tumor growth in control *Depdc5*^*ncl*^ mice, but greatly impaired tumor progression in *Depdc5*^*tko*^ mice (Fig. 7a–c). We next analyzed the tumor-infiltrating CD8^+^ T cell number in each group. Consistent with human CRC scRNA-seq data that CD8^+^ T cell infiltration in tumors is positively associated with the DEPDC5 level in CD8^+^ T cells (Fig. 1b), we found that there were small numbers of CD8^+^ T cells in tumors from *Depdc5*^*tko*^ mice compared with *Depdc5*^*ncl*^ mice (Fig. 7d, e). Furthermore, while DFO treatment had little impact on the number of tumor-infiltrating CD8^+^ T cells in *Depdc5*^*ncl*^ mice, this intervention significantly increased tumor infiltration of CD8^+^ T cells in *Depdc5*^*tko*^ mice (which almost reached the levels observed in *Depdc5*^*ncl*^ control animals; Fig. 7d, e). These data suggest that ferroptosis inhibition in *Depdc5*^*tko*^ mice can rescue the frequency and function of CD8^+^ T cells.

The above results predict that enhanced ferroptosis may impact anti-tumor immunity and could also limit the effectiveness of immune checkpoint blockade (ICB) therapy. To test this possibility, WT tumor-bearing mice were administered with GPX4 inhibitor RSL3 (or vehicle) daily via the I.P. injection route from day 5–7 after tumor inoculation. Anti-PD1 or isotype control antibodies were then administrated on day 10 and day 14, and tumor volumes were measured at the indicated time points (Fig. 7f, g). As expected, anti-PD1 antibody treatment significantly suppressed tumor growth compared with isotype control-treated mice (Fig. 7g). Surprisingly, compared to the vehicle-treated mice, RSL3-treated mice displayed more aggressive tumor growth in the presence of anti-PD1 antibody treatment (Fig. 7g), suggesting that inducting ferroptosis could impair the effectiveness of the anti-PD1-mediated ICB therapy.

## Discussion

DEPDC5 is a key component of the GATOR1 complex that plays a critical role in regulating mTORC1 activity when nutrient supplies are limited. *DEPDC5* has also been studied in the context of epilepsy since loss-of-function mutations in this gene have been implicated in multiple types of neuronal dysfunction. It was reported previously that there was a positive association between epilepsy and the rates of several types of cancers^61^, and some epilepsy patients had also weakened anti-infection immunity^62^. However, the molecular mechanism linking epilepsy and anti-tumor/anti-infection immunity has not been well studied to date. In the current report, we reveal a novel function of DEPDC5 in maintaining peripheral CD8^+^ T cell homeostasis and immunity by protecting this compartment from ferroptosis. Genetic ablation of *Depdc5* led to an aberrant increase of mTORC1 activity in CD8^+^ T cells, which in turn upregulated expression of transcription factor ATF4 and downstream target gene *Xdh*. Increased *Xdh* expression specifically encodes the short-form XO enzyme in T cells, thereby driving intracellular ROS production and lipid peroxidation, resulting in ferroptosis within the CD8^+^ pool. Inhibition of mTORC1 activity or ferroptosis suppression using VED/IFD restored CD8^+^ T cell homeostasis in *Depdc5*^*tko*^ mice. Treatment with DFO or Fer-1 was also able to suppress *Depdc5-*deficient CD8^+^ T cell death in vitro, suggesting that ferroptosis inhibition can directly promote the survival of cytotoxic lymphocytes. Importantly, inhibition of ferroptosis in vivo increased tumor infiltration by CD8^+^ T cells and extended survival of *Depdc5*^*tko*^ mice.

*Depdc5* is a well-known epilepsy disease gene. However, whether its mutation in T cells also contributes to epilepsy has not been studied. In our model, we did not observe any obvious signs of epilepsy or other neurological dysfunction in our *Depdc5*^*tko*^ mice during the course of our experiment. We could not rule out the long-term effect that may act indirectly on the neuronal system given the increased purine metabolism in *Depdc5-*deficient T cells, since a previous study by Fan et al. showed that stress could induce T cells to produce excessive xanthine, which was linked to depression^63^. However, our preliminary study did not reveal increased xanthine in our *Depdc5*^*tko*^ mice (data not shown).

Intriguingly, T cell-specific deletion of *Tsc1* has also been reported to augment mTORC1 activity and dysregulate peripheral T cell homeostasis without impacting thymic development^64^. Similar to our own findings in *Depdc5*^*tko*^ mice, *Tsc1* deletion impacted the CD8^+^ T cell pool more potently than other lymphocyte subsets, which the authors attributed to low expression of anti-apoptotic protein BCL-2. However, another study showed that deletion of *Tsc2* in T cells also led to reduced frequency of CD8^+^ but not CD4^+^ T cells in the periphery without changing the levels of BCL-2, BCL-XL, or cleaved caspase-3 but the authors suggested an altered cell proliferation and activation^65^.

Interestingly, the *Tsc1*-deficient CD8^+^ T cells had increased ROS production, resembling our own findings in *Depdc5*-deficient T cells, suggesting that although GATOR1 and TSC employ very different negative regulatory mechanisms to curb mTORC1 activity, the hyper-mTORC1-induced ferroptosis may be the common phenomena for impaired CD8^+^ T cell homeostasis when either the TSC or KICSTOR-GATOR1 complexes are disrupted. Other cell types with mTORC1 hyperactivation also had increased ROS, like *Tsc1*-deficient hematopoietic stem cells (HSC) and *Szt2*-deficient HSC, indicating increased ROS levels may be a general phenomenon in various types of cells with mTORC1 hyperactivation^66,67^. The current study also sheds important new light on the molecular mechanism by which elevated mTORC1 activity in CD8^+^ T cells can activate the ferroptosis process via XO-mediated ROS production.

Similar to the immune defect observed in *Depdc5*-deficient mice, T cell-specific deletion of anti-ferroptosis gene *Gpx4* can also impair homeostasis of the CD8^+^ subset while sparing the CD4^+^ compartment^23^. Like *Depdc5*-deficient and *Tsc2*-deficient mice, *Gpx4* deletion does not alter thymic T cell development, further suggesting that hyper-mTORC1-induced ferroptosis underpins the observed deficit in CD8^+^ T cell frequency and function. Consistently, inhibition of hyper-mTORC1 activity in either *Depdc5*-deficient or *Tsc2*-deficient mice reversed the CD8^+^ T cell defect^65^. While unclear if *Tsc1* or *Tsc2* deficiency in T cells influences ferroptosis, the elevated mTORC1 activity observed in these cells could also lead to increased purine metabolism. This may in turn upregulate the expression of genes including *Xdh*, leading to high levels of XO enzyme, ROS production, and ferroptosis of CD8^+^ T cells as observed in our study.

Why *Depdc5*-deficiency in T cells impacted CD8^+^ T cells more severely than CD4^+^ T cells is not fully understood. There could be several reasons. First, it could be due to the specific lipid ROS levels generated between *Depdc5*-deficient CD4^+^ and CD8^+^ T cells. As shown in Supplementary Fig. S3c, d, we found that although the mTORC1-ATF4-Xdh axis regulation was similar in CD4^+^ and CD8^+^ T cells, there were small but still significant differences in the lipid ROS levels between the CD4^+^ and CD8^+^ T cells, where the slightly higher lipid ROS level in *Depdc5*-deficient CD8^+^ T cells may count for their increased sensitivity to ferroptosis. Second, it was reported that CD8^+^ T cells had higher expression of iron transporter TFRC than CD4^+^ T cells^68^, suggesting that CD8^+^ T cells may have more iron, which could make CD8^+^ T cells more sensitive to ferroptosis induction than CD4^+^ T cells. Finally, it was shown before that deletion of the key ferroptosis inhibitor gene *Gpx4* in T cells also led to much more severe CD8^+^ T cell ferroptosis whereas it only marginally impacted CD4^+^ T cells^23^. Although no explanation was given for this difference, we believe that it could be due to the similar reasons as we discussed above.

High glucose levels can activate mTORC1 by suppressing the GATOR1-KICSTOR complex^11,12^. For people with hyperglycemia, such as diabetic patients, mTORC1 may be hyper-activated in peripheral T cells. At high levels of glucose, the DEPDC5-containing GATOR1-KICSTOR complex would be strongly suppressed, therefore resembling the features of CD8^+^ T cells from *Depdc5*^tko^ mice. Indeed, a previous study used a mouse diabetes mellitus model to show that blood CD8^+^ T cells but not CD4^+^ T cells were decreased in the setting of high blood glucose^69^. In agreement with this observation, a common problem in diabetic patients is frequent infections^70–72^, suggesting a possible defect in peripheral CD8^+^ T cell immunity. These data also suggest that hyperglycemia and *Depdc5*-deficiency may share certain pathological features, and our study further uncovers a number of potential strategies to rescue CD8^+^ T cell frequency and function in this context (including Vitamin E supplementation, iron restriction, or rapamycin treatment).

How elevated mTORC1 activity in diabetic patients might induce selective ferroptosis of CD8^+^ T cells has not been investigated. In our study of *Depdc5*-deficient CD8^+^ T cells, we observed that a key ferroptosis inducer downstream of mTORC1 is the transcription factor ATF4 (Fig. 6i). Intriguingly, it was previously reported that mTORC1 activation by high glucose levels increased translation of ATF4 protein, which could directly bind to the promoters of *Xdh* and other genes linked with purine metabolism, such as *Mthfd2*^46,59^. It is, therefore, possible that hyper-mTORC1 activity in CD8^+^ T cells, whether induced by high glucose or DEPDC5-deficiency, maybe a common mechanism of promoting glucose flux towards purine metabolism (via upregulation of ATF4-target genes including *Xdh* and *Mthfd2*). This could represent a useful strategy for combatting hyperglycemia, albeit at the expense of CD8^+^ T cell survival.

XOR belongs to the molybdenum-containing hydroxylase family. In addition to the oxidative metabolism of purines, XOR also has been identified as a moonlighting protein based on its ability to perform mechanistically distinct functions. For instance, XOR can function either as NADH-generating XDH protein or as ROS-generating XO protein^55,56^. Strikingly, the major protein form of *Xdh* detected in mouse CD8^+^ T cells was 125 kDa XO (Fig. 6g). Consistently, when XO-linked purine metabolism was increased in *Depdc5*-deficient CD8^+^ T cells, ROS levels also increased accordingly. These data may in part explain why elevated purine metabolism leads to divergent outcomes in different cell types.

Inducing cancer cell ferroptosis may represent a powerful strategy for cancer therapy^19,26,73^. However, systemic induction of ferroptosis in tumor tissue kills both cancer cells and cytotoxic lymphocytes^27,74,75^. In certain cancer models such as PDAC, systemic induction of ferroptosis can suppress tumor growth^26^, but other studies have observed superior anti-tumor immunity upon inhibition of ferroptosis (either via blockade of CD36-mediated uptake of oxidized lipids or by administration of direct inhibitors)^27,76^. In addition, it has also been reported that some immune populations including CD8^+^ T cells are more susceptible to ferroptosis than target cancer cells^29^. Furthermore, our analysis of human cancer sequencing data revealed better overall patient survival and more tumor infiltration by CD8^+^ T cells in individuals with high expression of *DEPDC5*, which this study has identified as an anti-ferroptosis gene (Fig. 1b, c).

Both CD4^+^ T cells and CD8^+^ T cells expressed *Depdc5* and displayed similar increases in mTORC1 activity upon deletion of this gene. However, only CD8^+^ T cell homeostasis was significantly impaired upon *Depdc5* deletion in vivo. While the underlying mechanism remains unclear, it is possible that the differing metabolic demands of each subset play an important role, e.g., distinct patterns of iron metabolism in CD4^+^ and CD8^+^ subsets. Intriguingly, genetic deletion of *Gpx4* in T cells induced a very similar phenotype, with CD8^+^ but not CD4^+^ subset being impaired in peripheral lymphoid organs^23^. Further studies will be needed to resolve how the DEPDC5-mTORC1 pathway influences ferroptosis differently in CD4^+^ and CD8^+^ T cells.

Other than DEPDC5, the impact of mutations in GATOR1-KICSTOR components on immune cell homeostasis and function remains poorly understood. Previous studies have shown that loss-of-function mutations in GATOR1-KICSTOR are associated with epilepsy and mTORC1 resistance to nutrient starvation^11,12,77^. Regarding the molecular mechanism described for T cells in our study, we believe that if DEPDC5 was deficient in other cells such as neurons, it would have a similar impact on those cells as it on T cells, at least for the purine metabolism. In this regard, we suspect that loss of function mutation of the GATOR1-axis in the neuronal system could impair the normal physiological function of the brain at least due to the defect in the mTORC1-mediated ROS regulation in neuron cells. Therefore, the strategies used here to rescue *Depdc5*-deficient CD8^+^ T cell function (Vitamin E supplementation, iron restriction, rapamycin treatment), may also prove effective for restoring cytotoxic lymphocyte responses in human patients with various mutations that impact this pathway.

## Materials and methods

### Mice

*Depdc5*-targeted (*Depdc5*^*f/f*^) mouse was generated by Cyagen Biosciences Inc. The Turbo Knockout targeting vector contained the long *Depdc5* homology arm with a *loxp* sequence inserted in the intron between exon 1 and exon 2 of *Depdc5* gene. This sequence encodes a 3× flag peptide in-frame fused with the translation initiation codon of *Depdc5* in exon 2 (thereby generating an *N*-terminal 3× flag-tagged DEPDC5 protein in vivo). The construct also included two self-deletion anchor sequence (SDA)-flanked neomycin resistance gene cassettes (*Neo*^r^) followed by a second *loxp* sequence immediately upstream of the short homology arm containing exon 3. Linearized vector was subsequently delivered to ES cells (C57BL/6) via electroporation, followed by drug selection, PCR screening, and southern blotting to confirm the successful targeting of the *Depdc5* gene. One of the successfully targeted ES cell lines was used to generate the targeted mouse line, and after one generation of self-crossing, the *Neo*^r^ gene was deleted by SDA during the spermatid stage to establish the *Depdc5*-*floxed* allele, which could be easily genotyped by PCR reaction using primers P1 (5′-GTAGCAGGAAAGCAAGATGACTTCC-3′) and P2 (5′-GATCCTGTGCTCTCATTTCCAACC-3′) to detect a 271 bp fragment from the WT (+) allele and a 341 bp fragment from the floxed allele (f).

*Cd4*-Cre transgenic mouse line was obtained from the Jackson Laboratory. All these mouse strains were bred and maintained at accredited animal facilities under specific pathogen-free conditions in individually ventilated cages on a strict 12 h day/night cycle with a normal chow diet. Unless otherwise indicated, 6–10-week-old and sex-matched mice were used in all assays. All animal experiments were conducted in accordance with local guidelines for the use and care of laboratory animals as provided by Shanghai Jiao Tong University School of Medicine Institutional Animal Care and Use Committees (IACUC).

### CD8^+^ T cell isolation and treatment with cell death inhibitors in vitro

Single-cell suspensions were freshly prepared from LN with indicated mice. 5 × 10^5^ cells were seeded in a round 96-wel1 plate (5 × 10^5^ cells/well), which is pre-coated with anti-CD3 (5 μg/mL; from BD), and treated with indicated inhibitors (10 μM Z-VAD-FMK, 10 μM Necrostatin-1, 5 μM Ferrostatin-1 or 100 μM Deferoxamine) in RPMI Medium 1640 (Gibco, cat# 11875-093) containing 10% FBS and 1% Penicillin-Streptomycin culture at 37 °C for indicated time for 4 or 6 h.

The cell pellets were collected by centrifuge at 2000 rpm for 5 min at 4 °C and the supernatant was discarded, followed by cold PBS wash once, centrifuge at 2000 rpm for 5 min at 4 °C. Then the pellets were stained with Sytox green viability or Live/Dead stain dye, anti-B220 antibody, or anti-CD8 antibody. The ratios of dead cells were quantified by analyzing sytox green or Live/Dead stain-positive cell percentages in indicated CD8^+^ T or B cells through flow cytometry.

### Xenograft tumor model and treatment

6-week-old *Depdc5*^*ncl*^ and *Depdc5*^*tko*^ mice were inoculated with 5 × 10^6^ MC38 colon tumor cells, and tumor volumes were measured 10 days after tumor inoculation (tumor volume equals length × width × height).

For the CD8^+^ T cells depletion experiment, 6-week-old *Depdc5*^*ncl*^ and *Depdc5*^*tko*^ mice were inoculated with 5 × 10^5^ MC38 colon tumor cells, the control group I.P. injection treated with PBS, the CD8^+^ T depletion group treated as I.P. injection anti-CD8 antibody (250 μg per mouse per time) at day 3, day 6, day 9 after tumor inoculation. Tumor volumes were measured 10 days after tumor inoculation, volume = length × width × height (mm). For the DFO-treated tumor model, *Depdc5*^*ncl*^ and *Depdc5*^*tko*^ mice were treated daily with 200 μg/kg DFO by I.P. injection from day 0 after tumor inoculation. Mice were killed after tumor volume reached 1000 mm^3^. For the anti-PD1 treatment experiment, 6-week-old female WT mice were inoculated with 5 × 10^5^ KLN205 tumor cells, the control group I.P. injection treated with vehicle or RSL3 (2.2 mg/kg) from day 5 to day 7 after KLN205 celll inoculation, then mice were I.P. injection treated with isotype or anti-PD1 antibody (100 μg per mouse per time) at day 10 and day 14 after tumor inoculation. Tumor volumes were measured 10 days after tumor inoculation, volume = length × width × height (mm). Mice were sacrificed on day 20.

### Rapamycin treatment

Adult *Depdc5*^*ncl*^ and *Depdc5*^*tko*^ mice (4–6 weeks old) were constitutively treated with 100 μg/kg/day rapamycin via I.P. injection for 4 weeks, the control group of *Depdc5*^*ncl*^ and *Depdc5*^*tko*^ mice (4–6 weeks old) were I.P. injection with 100 μL PBS every day for 4 weeks.

### High Vitamin E and iron-free diet treatment

Adult *Depdc5*^*ncl*^ and *Depdc5*^*tko*^ mice (6-week-old) were divided into two groups. One group still fed on a normal chow diet as before, and the other group changed the diet to a high Vitamin E or iron-free diet for 4 weeks. Vitamin E diet was reformed from the AIN-76A diet formula via adding additional Vitamin E to the diet to a final concentration of 1000 mg/kg Vitamin E, Iron-free diet was reformed from TD.80396 diet formula via adding no ferric citrate by Jiangsu Xietong Pharmaceutical Bio-engineering Co., Ltd.

### RNA extraction for RNA-seq and RT-qPCR

6-week-old *Depdc5*^*ncl*^ and *Depdc5*^*tko*^ mice were used for CD8^+^ T cell isolation and RNA preparation. Primary CD8^+^ T cells were isolated from the spleen of each mouse by using a CD8^+^ T cells negative isolate kit (from STEMCELL Technologies, cat# 19853a) and then further purified by flow cytometry cell sorting based on CD8 marker. All sorted cells were lysed with TRIzol^TM^ Reagent (from Life Technologies, cat# 15596018) at –80 °C overnight respectively; then, total RNA was extracted from the TRIzol^TM^ Reagent according to standard protocols. Then the total RNA of each sample was used for RNA-seq.

For RT-qPCR analysis, after total RNA was extracted, the RNA was immediately reverse-transcribed into cDNA using the PrimeScript™ RT reagent Kit (Perfect Real Time) (Takara, cat# RR037A). RT-qPCR was subsequently performed using SYBR Green Real-time PCR Master Mix (Takara, cat# RR036A) together with a ViiA7 Real-Time PCR System (Applied Biosystems). All primers were purchased from Sangon Biotech.

### Flow cytometry analysis and fluorescence-activated cell sorting (FACS)

PBMCs were obtained from patients with epilepsy at Shanghai Children’s Medical Center, Shanghai Jiao Tong University School of Medicine. This project was approved by the local ethics committee (SCMCIRB— K2019026-2). Informed consent was obtained from each patient, and the study protocol was approved by the Clinical Research Ethics Committee of Shanghai Children’s Medical Center and complied with all relevant ethical regulations.

For mouse lymphocyte surface staining, single-cell suspensions were stained with fluorophore-conjugated anti-mouse antibodies diluted in 50 μL PBS containing 2% FBS on ice for 30 min in the dark. Cells were washed once with 1× PBS containing 2% FBS and re-suspended in 1× PBS containing 2% FBS for flow cytometry or cell sorting. Normally, Fixable Viability Stain 700 (AF700, BD) or L/D PE-CF594 (BD), FITC anti-mouse CD3 (17A2, Biolegend), Brilliant Violet 421™ anti-mouse CD4 (GK1.5, Biolegend), Brilliant Violet 421™ anti-mouse CD8a (53-6.7, Biolegend), APC-eFluor 780 anti-mouse CD4 (RM4-5, eBioscience), APC-eFluor 780 anti-mouse CD8 (53-6.7, eBioscience) and PE anti-mouse B220 (RA3-6B2, eBioscience) and Alexa Fluor® 647 anti-mouse CD19 (6D5, Biolegend) were used for staining.

### Intracellular S6-pS235/236 and S6K-pT389 staining

For intracellular S6-pS235/236 and S6K-pT389 staining, after stained with live/dead Fixable Viability and surface marker CD4, CD8 staining and 200 μL cold FACS wash, the pellets were suspended with 200 μL BD Phosflow Fix Buffer I, incubated at 4 °C for 1 h, centrifuged at 2000 rpm at 4 °C for 5 min and the supernatant was discarded. Then room temperature (RT) 1× Perm/Wash Buffer I (dilute with distilled water) was used to wash the pellets twice, and then cells were stained in 50 μL 1× Perm/Wash Buffer I, with anti-Phospho-p70 S6 Kinase (Thr389) (CST, cat# 9234) rabbit mAb or anti-Phospho-S6 Ribosomal Protein (Ser235/236) (CST, cat# 2211 S), RT for 60 min. After washed twice with 200 μL 1× Perm/Wash Buffer I, the cells were then stained in 50 μL 1× Perm/Wash Buffer I with goat anti-rabbit IgG H&L (Alexa Fluor® 488, Abcam, cat# ab150081) or goat anti-rabbit IgG H&L (Alexa Fluor® 647, Abcam, cat# ab150087) from Abcam, RT for 30 min. The cells were washed twice with 1× Perm/Wash Buffer I and then collected the data on LSRFortessa X-20.

### Immunoblotting analysis

Immunoblotting analysis was performed using the following antibodies: anti-Phospho-p70 S6 Kinase (Thr398) (cat# 9324), anti-p70 S6 Kinase (cat# 9202), anti-p44/42 (Erk1/2) (cat# 9102), anti-ATF-4 (cat# 11815), anti-p38 (cat# 8690) from CST, anti-Flag (Sigma-Aldrich, cat#11815), anti-Xdh (Proteintech, cat# 55156-1-AP), anti-HSP90 (Abclonal, cat# A5027). Briefly, protein concentration in samples was quantified by Quick Start™ Bradford 1× Dye Reagent (BIO-RAD, cat# 5000205); then the samples were equally loaded for electrophoresis and membrane transfer. The membrane was blocked with 5% (m/v) milk in TBST (0.1% Tween 20) RT for 30 min before being incubated with primary antibody overnight at 4 °C. The membrane was washed three times with TBST and incubated with the corresponding secondary antibody RT for 1 h. The membrane was washed three times before exposure using Immobilon Western Chemilum HRP Substrate (Millipore Sigma).

### Detection of Ki-67 level

Fresh cell suspension from the spleen was firstly stained with Fixable Viability Stain 700 (BD), anti-CD4 antibody (Biolegend) and anti-CD8 antibody (eBioscience); then PE Mouse Anti-Ki-67 Set (BD Pharmingen™, cat# 556027) was used to detect Ki-67 level in T cells.

### Detection of lipid peroxidation

One million cells from the spleen or LN were seeded in a Round 96-well plate (10^6^ cells/well) with anti-CD3 (5 μg/mL; from BD) and were loaded with Bodipy^TM^ 581/591 (2 μM, from Invitrogen) in RPMI Medium 1640 (Gibco, cat# 11875-093) containing 10% FBS and 1% Penicillin-Streptomycin culture at 37 °C for 2 h or 4 h. Then the cell pellets were collected by centrifuge at 2000 rpm for 5 min at 4 °C and discard the supernatant, followed by cold PBS wash twice, centrifuged at 2000 rpm for 5 min at 4 °C, then stained with Fixable Viability Stain 700 and surface marker CD4 and CD8. BODIPY emission was recorded in the FITC panel using LSRFortessa X-20.

### Detection of ROS staining by flow cytometry

Cellular reactive oxidative species were detected by CM-H_2_DCFDA (Life Technologies, cat# C6827). Mouse splenocytes were seeded in a Round 96-well plate with 10 μM allopurinol treated for 2 h or not, then stained with 5 μM CM-H2DCFDA in PBS (Life Technologies) at 37 °C for 15 min. After incubation, the cells were collected by centrifuge at 1500 rpm for 5 min at 4 °C, followed by cold PBS wash twice and staining surface marker; ROS emission was recorded on FITC panel fluorescence levels by flow cytometry.

### RNA-seq and analysis

CD8^+^ T cells RNA extracted from 4 pairs of 6-week-old female mice splenocytes were used for RNA-seq. The raw reads were aligned to the mouse reference genome using the HISAT2 alignment program. The R package DESeq2 was used to normalize the raw counts and identify differentially expressed genes. Significant DEGs are defined as *P* value < 0.05 and |log_2_FC| ≥ 0.5. GO enrichment analysis was performed by the R package cluster Profiler. Kyoto Encyclopedia of Genes and Genomes pathway (KEGG) enrichment analysis was performed by Gene Set Enrichment Analysis (GSEA), where the differentially expressed genes identified as described above were supplied as the input for genes by function and enrich GO and enrich KEGG, respectively.

### Statistical analysis

Statistical analysis was performed by using GraphPad Prism 6 software. *P* values were calculated with unpaired Student’s *t*-test. All error bars were represented as means ± SEM. *P* values of less than 0.05 were considered statistically significant. *P < 0.05, **P < 0.01, ***P < 0.001, ****P < 0.0001.

### Supplementary Information

Supplementary Information

## Data Availability

All the data supporting this paper were provided in the main text and Supplementary Information of this article. The raw files of bulk RNA-seq were stored at CNGB Sequence Archive (CNSA) of China National GeneBank DataBase (CNGBdb, https://db.cngb.org) under login number CNP0004390 (Through the URL: https://db.cngb.org/search/?q=CNP0004390). The processed public bulk RNA-seq dataset were downloaded from Gene Expression Omnibus (GEO, https://www.ncbi.nlm.nih.gov/geo/), including GSE23878^32^, GSE37364^33^, GSE18105^34^, GSE21510^35^, and GSE17537^36^; and public normalized gene expression by fragments per kilobase of exon model per million reads mapped (FPKM) of pan-Cancer were downloaded from TCGA data portal (http://gdac.broadinstitute.org/). All the scripts, codes, and other materials are available from the authors upon request.

## References

[1] Philip M, Schietinger A (2022). CD8(+) T cell differentiation and dysfunction in cancer. Nat. Rev. Immunol..

[2] Chen Y (2023). Regulation of CD8(+) T memory and exhaustion by the mTOR signals. Cell. Mol. Immunol..

[3] Vesely MD, Zhang T, Chen L (2022). Resistance Mechanisms to Anti-PD Cancer Immunotherapy. Annu. Rev. Immunol..

[4] Kawabe T, Yi J, Sprent J (2021). Homeostasis of Naive and Memory T Lymphocytes. Cold Spring Harb. Perspect. Biol..

[5] Ramanathan S (2009). Cytokine synergy in antigen-independent activation and priming of naive CD8(+) T lymphocytes. Crit. Rev. Immunol..

[6] Lee SW, Lee GW, Kim HO, Cho JH (2023). Shaping Heterogeneity of Naive CD8(+) T Cell Pools. Immune Netw..

[7] Gonzalez A, Hall MN (2017). Nutrient sensing and TOR signaling in yeast and mammals. EMBO J..

[8] Ruan C (2019). Sin1-mediated mTOR signaling in cell growth, metabolism and immune response. Natl. Sci. Rev..

[9] Liu GY, Sabatini DM (2020). mTOR at the nexus of nutrition, growth, ageing and disease. Nat. Rev. Mol. Cell Biol..

[10] Bar-Peled L (2013). A Tumor suppressor complex with GAP activity for the Rag GTPases that signal amino acid sufficiency to mTORC1. Science.

[11] Wolfson RL (2017). KICSTOR recruits GATOR1 to the lysosome and is necessary for nutrients to regulate mTORC1. Nature.

[12] Peng M, Yin N, Li MO (2017). SZT2 dictates GATOR control of mTORC1 signalling. Nature.

[13] Ishida S (2013). Mutations of DEPDC5 cause autosomal dominant focal epilepsies. Nat. Genet.

[14] Dibbens LM (2013). Mutations in DEPDC5 cause familial focal epilepsy with variable foci. Nat. Genet..

[15] Baldassari S (2019). The landscape of epilepsy-related GATOR1 variants. Genet. Med..

[16] Jiang X, Stockwell BR, Conrad M (2021). Ferroptosis: mechanisms, biology and role in disease. Nat. Rev. Mol. Cell Biol..

[17] Li S, Wang QJ, Su B (2021). mTOR-mediated cell death and infection. Infect. Microbes Dis..

[18] Dixon SJ (2012). Ferroptosis: An iron-dependent form of nonapoptotic cell death. Cell.

[19] Stockwell BR (2017). Ferroptosis: A regulated cell death nexus linking metabolism, redox biology, and disease. Cell.

[20] Hassannia B, Vandenabeele P, Vanden Berghe T (2019). Targeting ferroptosis to iron out cancer. Cancer Cell.

[21] Conrad M, Pratt DA (2019). The chemical basis of ferroptosis. Nat. Chem. Biol..

[22] Zhang X (2022). Vitamin E exerts neuroprotective effects in Pentylenetetrazole kindling epilepsy via suppression of ferroptosis. Neurochem. Res..

[23] Matsushita M (2015). T cell lipid peroxidation induces ferroptosis and prevents immunity to infection. J. Exp. Med..

[24] Su Y (2020). Ferroptosis, a novel pharmacological mechanism of anti-cancer drugs. Cancer Lett..

[25] Qiu Y, Cao Y, Cao W, Jia Y, Lu N (2020). The application of ferroptosis in diseases. Pharmacol. Res..

[26] Badgley MA (2020). Cysteine depletion induces pancreatic tumor ferroptosis in mice. Science.

[27] Ma X (2021). CD36-mediated ferroptosis dampens intratumoral CD8(+) T cell effector function and impairs their antitumor ability. Cell Metab..

[28] Dai E (2020). Ferroptotic damage promotes pancreatic tumorigenesis through a TMEM173/STING-dependent DNA sensor pathway. Nat. Commun..

[29] Drijvers JM (2021). Pharmacologic screening identifies metabolic vulnerabilities of CD8(+) T Cells. Cancer Immunol. Res..

[30] Samanta D (2022). DEPDC5-related epilepsy: A comprehensive review. Epilepsy Behav..

[31] Zhang L (2020). Single-cell analyses inform mechanisms of myeloid-targeted therapies in colon cancer. Cell.

[32] Uddin S (2011). Genome-wide expression analysis of Middle Eastern colorectal cancer reveals FOXM1 as a novel target for cancer therapy. Am. J. Pathol..

[33] Valcz G (2014). Myofibroblast-derived SFRP1 as potential inhibitor of colorectal carcinoma field effect. PloS One.

[34] Matsuyama T (2010). MUC12 mRNA expression is an independent marker of prognosis in stage II and stage III colorectal cancer. Int. J. Cancer.

[35] Tsukamoto S (2011). Clinical significance of osteoprotegerin expression in human colorectal cancer. Clin. Cancer Res..

[36] Freeman TJ (2012). Smad4-mediated signaling inhibits intestinal neoplasia by inhibiting expression of β-catenin. Gastroenterology.

[37] Karlsson M (2021). A single-cell type transcriptomics map of human tissues. Sci. Adv..

[38] Liao Y (2020). Inflammation mobilizes copper metabolism to promote colon tumorigenesis via an IL-17-STEAP4-XIAP axis. Nat. Commun..

[39] Sawada S, Scarborough JD, Killeen N, Littman DR (1994). A lineage-specific transcriptional silencer regulates CD4 gene expression during T lymphocyte development. Cell.

[40] Wang X (2011). MEKK3 regulates IFN-gamma production in T cells through the Rac1/2-dependent MAPK cascades. J. Immunol..

[41] Drummen GP, van Liebergen LC, Op den Kamp JA, Post JA (2002). C11-BODIPY(581/591), an oxidation-sensitive fluorescent lipid peroxidation probe: (micro)spectroscopic characterization and validation of methodology. Free Radic. Biol. Med..

[42] Kuvibidila S, Dardenne M, Savino W, Lepault F (1990). Influence of iron-deficiency anemia on selected thymus functions in mice: thymulin biological activity, T-cell subsets, and thymocyte proliferation. Am. J. Clin. Nutr..

[43] Wang X, Proud CG (2009). Nutrient control of TORC1, a cell-cycle regulator. Trends Cell Biol..

[44] Kolev M (2015). Complement regulates nutrient influx and metabolic reprogramming during Th1 cell responses. Immunity.

[45] Ben-Sahra I, Howell JJ, Asara JM, Manning BD (2013). Stimulation of de novo pyrimidine synthesis by growth signaling through mTOR and S6K1. Science.

[46] Ben-Sahra I, Hoxhaj G, Ricoult SJH, Asara JM, Manning BD (2016). mTORC1 induces purine synthesis through control of the mitochondrial tetrahydrofolate cycle. Science.

[47] Latunde-Dada GO (2017). Ferroptosis: Role of lipid peroxidation, iron and ferritinophagy. Biochim. Biophys. Acta Gen. Subj..

[48] Liu J, Kang R, Tang D (2021). Signaling pathways and defense mechanisms of ferroptosis. FEBS J..

[49] Sies H, Jones DP (2020). Reactive oxygen species (ROS) as pleiotropic physiological signalling agents. Nat. Rev. Mol. Cell Biol..

[50] Lei G, Zhuang L, Gan B (2022). Targeting ferroptosis as a vulnerability in cancer. Nat. Rev. Cancer.

[51] Chen X, Li J, Kang R, Klionsky DJ, Tang D (2021). Ferroptosis: machinery and regulation. Autophagy.

[52] Bersuker K (2019). The CoQ oxidoreductase FSP1 acts parallel to GPX4 to inhibit ferroptosis. Nature.

[53] Doll S (2019). FSP1 is a glutathione-independent ferroptosis suppressor. Nature.

[54] Song X (2016). FANCD2 protects against bone marrow injury from ferroptosis. Biochem. Biophys. Res. Commun..

[55] Nishino T, Okamoto K, Eger BT, Pai EF, Nishino T (2008). Mammalian xanthine oxidoreductase - mechanism of transition from xanthine dehydrogenase to xanthine oxidase. FEBS J..

[56] Chung HY (1997). Xanthine dehydrogenase/xanthine oxidase and oxidative stress. Age.

[57] Dalbeth N, Merriman TR, Stamp LK (2016). Gout. Lancet.

[58] Selvarajah B (2019). mTORC1 amplifies the ATF4-dependent de novo serine-glycine pathway to supply glycine during TGF-beta1-induced collagen biosynthesis. Sci. Signal..

[59] Dasgupta S (2018). Metabolic enzyme PFKFB4 activates transcriptional coactivator SRC-3 to drive breast cancer. Nature.

[60] Park Y, Reyna-Neyra A, Philippe L, Thoreen CC (2017). mTORC1 balances cellular amino acid supply with demand for protein synthesis through post-transcriptional control of ATF4. Cell Rep..

[61] Kaae J, Carstensen L, Wohlfahrt J, Melbye M, Allison Boyd H (2014). Epilepsy, anti-epileptic medication use and risk of cancer. Int. J. Cancer.

[62] Aarli JA (2000). Epilepsy and the immune system. Arch. Neurol..

[63] Fan KQ (2019). Stress-induced metabolic disorder in peripheral CD4(+) T cells leads to anxiety-like behavior. Cell.

[64] Yang K, Neale G, Green DR, He W, Chi H (2011). The tumor suppressor Tsc1 enforces quiescence of naive T cells to promote immune homeostasis and function. Nat. Immunol..

[65] Pollizzi KN (2015). mTORC1 and mTORC2 selectively regulate CD8(+) T cell differentiation. J. Clin. Invest..

[66] Chen C (2008). TSC-mTOR maintains quiescence and function of hematopoietic stem cells by repressing mitochondrial biogenesis and reactive oxygen species. J. Exp. Med..

[67] Yin N (2022). SZT2 maintains hematopoietic stem cell homeostasis via nutrient-mediated mTORC1 regulation. J. Clin. Invest..

[68] Motamedi M, Xu L, Elahi S (2016). Correlation of transferrin receptor (CD71) with Ki67 expression on stimulated human and mouse T cells: The kinetics of expression of T cell activation markers. J. Immunol. Methods.

[69] Muller YD (2011). Immunosuppressive effects of streptozotocin-induced diabetes result in absolute lymphopenia and a relative increase of T regulatory cells. Diabetes.

[70] Lipsky BA (2020). Guidelines on the diagnosis and treatment of foot infection in persons with diabetes (IWGDF 2019 update). Diabetes Metab. Res. Rev..

[71] Kim SY (2021). Genetic diagnosis of infantile-onset epilepsy in the clinic: Application of whole-exome sequencing following epilepsy gene panel testing. Clin. Genet..

[72] Pearson-Stuttard J (2021). Type 2 diabetes and cancer: an umbrella review of observational and mendelian randomization studies. Cancer Epidemiol. Biomarkers Prev..

[73] Song J (2021). The deubiquitinase OTUD1 enhances iron transport and potentiates host antitumor immunity. EMBO Rep..

[74] Friedmann Angeli JP, Krysko DV, Conrad M (2019). Ferroptosis at the crossroads of cancer-acquired drug resistance and immune evasion. Nat. Rev. Cancer.

[75] Jia M (2020). Redox homeostasis maintained by GPX4 facilitates STING activation. Nat. Immunol..

[76] Xu S (2021). Uptake of oxidized lipids by the scavenger receptor CD36 promotes lipid peroxidation and dysfunction in CD8(+) T cells in tumors. Immunity.

[77] Basel-Vanagaite L (2013). Biallelic SZT2 mutations cause infantile encephalopathy with epilepsy and dysmorphic corpus callosum. Am. J. Hum. Genet..

