# Supplementary material for: DEPDC5 protects CD8+ T cells from ferroptosis by limiting mTORC1-mediated purine catabolism
Source: Cell Discov. 2024 May 20;10:53. doi: 10.1038/s41421-024-00682-z (PMC11102918; doi:10.1038/s41421-024-00682-z)
Supplement: Supplementary file 1 — Supplementary Information [file 41421_2024_682_MOESM1_ESM.pdf]

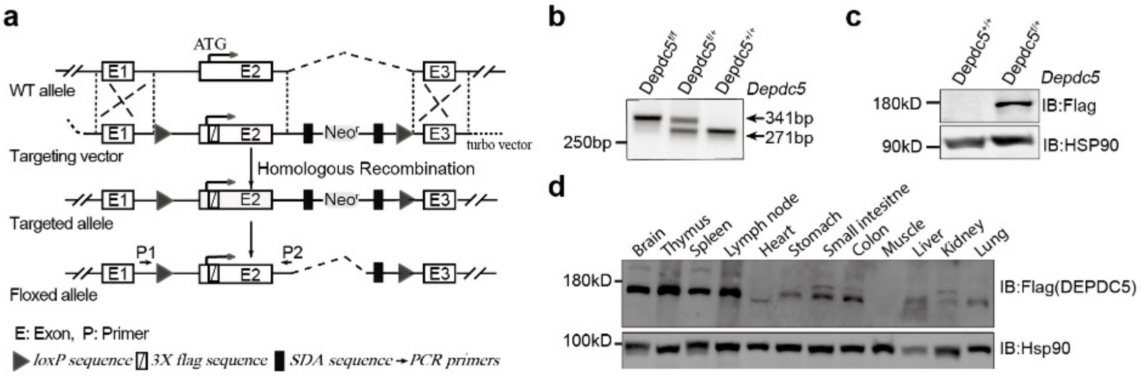

Fig. S1 DEPDC5 is highly expressed in lymphoid tissues.

a Diagram illustrating the exon 1-3 region of wild type *Depdc5* allele (WT allele), *Depdc5* targeting vector (Targeting vector), *Depdc5* targeted allele (Targeted allele), and *Depdc5* floxed allele (floxed allele). E1, E2, E3: *Depdc5* exon 1, exon 2, and exon 3; *loxP*: *Loxp* sequence; 3 × flag sequence: MDYKDHDGDYKDHDIDYKDDDDK; SDA: self-deletion anchor sequences; Neo^r: neomycin resistance gene; HR: homologous recombination; PCR primers P1 and P2: primers used to genotype the floxed allele.

b DNA prepared from the toes of *Depdc5*$^{f/f}$, *Depdc5*$^{f/+}$, and *Depdc5*$^{+/+}$ mice were used for PCR reaction with P1 and P2 primers as indicated in panel (**a**), which amplified 341 bp and 271 bp fragments from *Depdc5* floxed and wild type alleles, respectively.

c Brain tissue lysates from *Depdc5*$^{+/+}$ and *Depdc5*$^{f/+}$ mice were analyzed by immunoblotting with anti-flag antibody. Anti-HSP90 immunoblotting served as a loading control.

d Anti-flag antibody immunoblotting of brain, thymus, spleen, lymph nodes, heart, stomach, small intestine, colon, muscle, liver, kidney, and lung tissue lysates from *Depdc5*$^{f/f}$ mice. Anti-HSP90 immunoblotting served as a loading control.

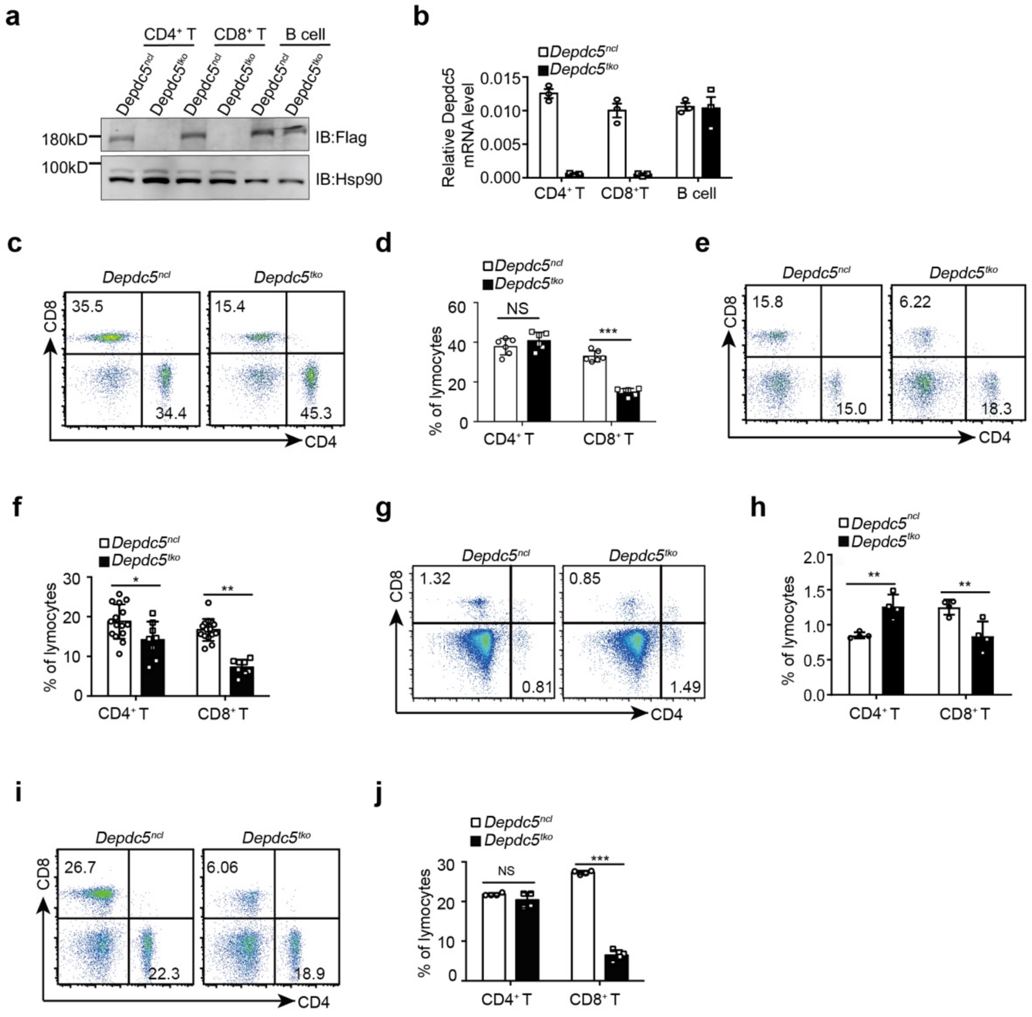

Fig.S2 DEPDC5 is required for maintaining the CD8$^+$ compartment.

a CD4$^+$ T cells, CD8$^+$ T cells, and B cells isolated from *Depdc5^{ncl}* and *Depdc5^{tko}* mice were analyzed by immunoblotting with anti-flag antibody. Anti-HSP90 immunoblotting served as a loading control.

b RT-qPCR analysis of *Depdc5* deletion in T cells from *Depdc5^{ncl}* and *Depdc5^{tko}* mice. Total RNA from CD4$^+$ T cells, CD8$^+$ T cells, and B cells isolated from *Depdc5$^{f/f}$* and *Depdc5^{tko}* mice was used for RT-qPCR analysis. Relative *Depdc5* mRNA level was normalized to *Actin* mRNA level.

c Flow cytometry analysis of LN CD4$^+$ and CD8$^+$ T cells from *Depdc5^{ncl}* and *Depdc5^{tko}* mice using anti-mouse CD4 and CD8 antibodies. Numbers in each quadrant show percentage of the gated populations.

d Summary bar graph showing CD4$^+$ and CD8$^+$ T cells as percentages of total LN

lymphocytes from 6-week-old *Depdc5^ncl^* and *Depdc5^tko^* mice (*n* = 6).

e Flow cytometry analysis of peripheral blood $CD4^+$ and $CD8^+$ T cells from $Depdc5^{ncl}$ and $Depdc5^{tko}$ mice after staining with anti-mouse CD4 and CD8 antibodies. Numbers in each quadrant show percentage of the gated populations.

f Summary bar graphs showing $CD4^+$ and $CD8^+$ T cell percentages in the blood of $Depdc5^{ncl}$ mice (n = 15) and $Depdc5^{tko}$ mice (n = 8).

g Flow cytometry analysis of bone marrow $CD4^+$ and $CD8^+$ T cells from $Depdc5^{ncl}$ and $Depdc5^{tko}$ mice after staining with anti-mouse CD4 and CD8 antibodies. Numbers in each quadrant show percentage of the gated populations.

h Summary bar graphs showing $CD4^+$ and $CD8^+$ T cell percentages in the bone marrow of $Depdc5^{ncl}$ mice (n = 4) and $Depdc5^{tko}$ mice (n = 4).

i Flow cytometry analysis of splenic $CD4^+$ and $CD8^+$ T cells from 6-month-old $Depdc5^{ncl}$ and $Depdc5^{tko}$ mice after staining with anti-mouse CD4 and CD8 antibodies. Numbers in each quadrant show percentage of the gated populations.

j Summary bar graph of splenic $CD4^+$ and $CD8^+$ T cell percentages in 6-month-old $Depdc5^{ncl}$ and $Depdc5^{tko}$ mice (n = 4). Error bars indicate mean ± SEM (NS, not significant, * $P < 0.05$, ** $P < 0.01$, *** $P < 0.001$ by unpaired Student's *t* test).

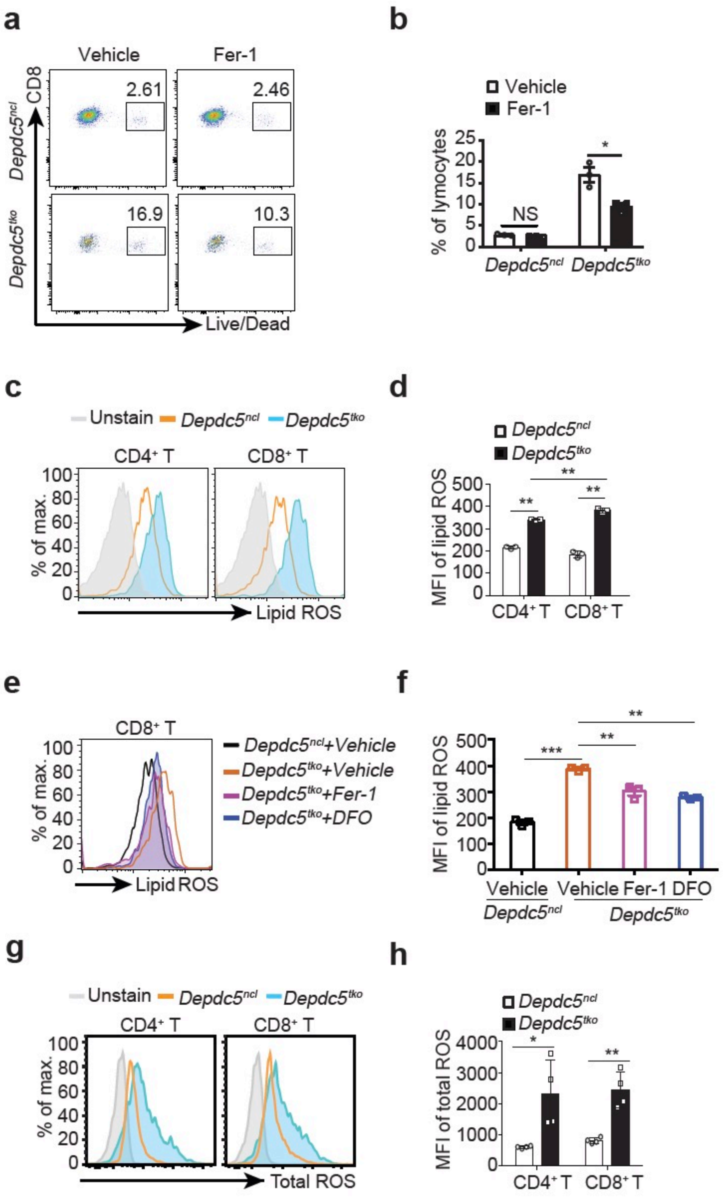

Fig.S3 *Depdc5*-deficient CD8$^+$ T cells display increased lipid peroxidation.

a Flow cytometry analysis of CD8$^+$ T cell death with vehicle or Fer-1 treatment. LN cells from *Depdc5^{ncl}* and *Depdc5^{tko}* mice were cultured in vitro in vehicle or with Fer-1 for 6 h, followed by flow cytometry analysis with an anti-CD8 antibody for CD8$^+$ T cells.

b Summary bar graph showing the percentage of dead CD8$^+$ T cells with vehicle or Fer-1 treatment as described in panel (**a**) (n = 3).

c Representative histograms showing lipid ROS levels in CD4$^+$ and CD8$^+$ T cells from *Depdc5^{ncl}* and *Depdc5^{tko}* mice. LN lymphocytes from *Depdc5^{ncl}* and *Depdc5^{tko}* mice were cultured in vitro for 4 h in the presence of 2 μM BODIPY™ 581/591 C11. Cells were washed twice with PBS before staining with a fixable viability dye and anti-mouse CD4

and CD8 antibodies for flow cytometry analysis.

d Summary bar graph showing lipid ROS levels in $CD4^+$ and $CD8^+$ T cells treated as described in panel (**c**) (*n* = 3).

e A representative histogram showing lipid ROS levels in $CD8^+$ T cells from *Depdc5*^{tko} mice treated with vehicle, 5 μM Fer-1, or 100 μM DFO, respectively.

f A summary bar graph showing lipid ROS levels in $CD8^+$ T cells treated as described in panel (**e**) (*n* = 3).

g Representative histograms showing total ROS levels in $CD4^+$ and $CD8^+$ T cells from *Depdc5*^{ncl} and *Depdc5*^{tko} mice. Splenocytes from *Depdc5*^{ncl} and *Depdc5*^{tko} mice were first stained with a fixable viability dye, anti-mouse CD4 and CD8 antibodies, then incubated in medium containing H_2DCFDA (5 μM) for 15 min at 37 ℃. Cells were washed twice in PBS before immediate flow cytometry analysis.

h Bar graph showing total ROS levels in $CD4^+$ and $CD8^+$ T cells from *Depdc5*^{ncl} and *Depdc5*^{tko} mice (*n* = 4). Error bars indicate mean ± SEM (* $P < 0.05$, ** $P < 0.01$ by unpaired Student's *t* test).

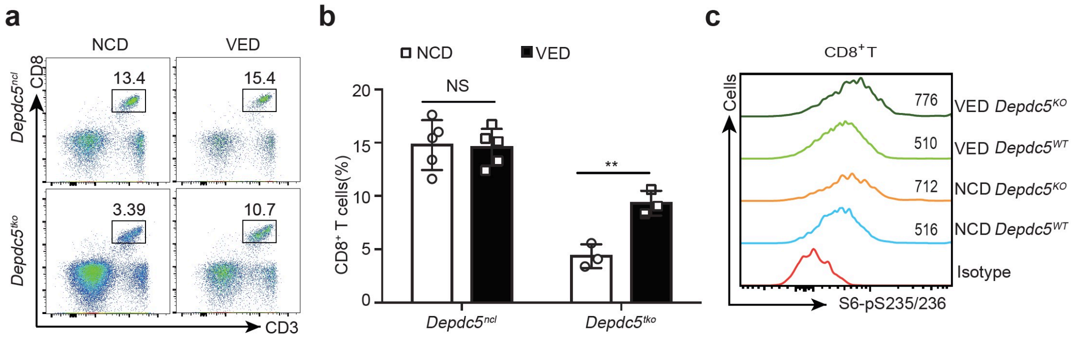

Fig.S4 Detection of blood CD8$^+$ T cells and S6 phosphorylation in CD8$^+$ T cells from NCD and VED-treated *Depdc5^{ncl}* and *Depdc5^{tko}* mice.

a Flow cytometry analysis of blood CD3$^+$CD8$^+$ T cells from *Depdc5^{ncl}* and *Depdc5^{tko}* mice fed with NCD or VED for 4 weeks. Numbers in quadrants indicate percentage of the gated subsets.

b Summary bar graph showing blood CD8$^+$ T cell percentages in *Depdc5^{ncl}* and *Depdc5^{tko}* mice as described in panel **a** (*Depdc5^{ncl}* mice, n = 6; *Depdc5^{tko}* mice, n = 3).

c A histogram showing phosphorylated S6-S235/236 levels in CD8$^+$ T cells from *Depdc5^{ncl}* and *Depdc5^{tko}* mice fed with NCD and VED. Splenocytes from *Depdc5^{ncl}* and *Depdc5^{tko}* mice fed with NCD and VED were analyzed by flow cytometry with antibodies against CD8 and S6-S235/236 phosphorylation. The numbers on the right indicate the mean fluorescence intensity. Error bars indicate mean ± SEM. (NS, not significant, ** $P <$ 0.01 by unpaired Student's t test).

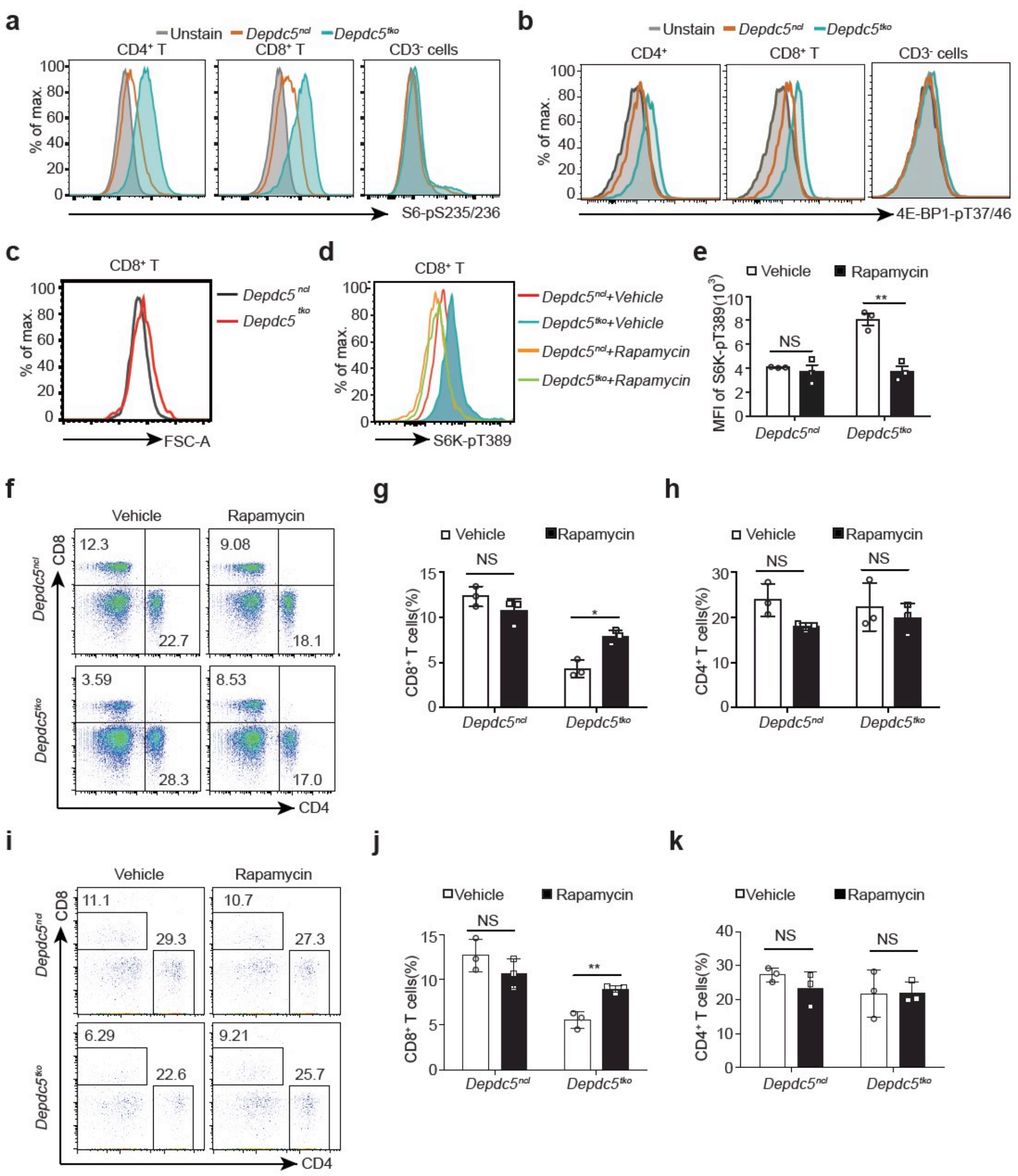

Fig.S5 Determination of CD8^{+} T cell percentage and mTORC1 downstream substrates phosphorylation in *Depdc5*^{ncl} and *Depdc5*^{tko} mice treated with rapamycin or vehicle only.

a Representative histograms showing phosphorylated S6-S235/236 levels in CD4^{+} T cells, CD8^{+} T cells, and CD3 negative cells from *Depdc5*^{ncl} and *Depdc5*^{tko} mice. Splenocytes from *Depdc5*^{ncl} and *Depdc5*^{tko} mice were stained with fixable viability dye and anti-mouse antibodies against CD3, CD4, CD8, and S6-S235/236 phosphorylation prior to analysis by flow cytometry.

b Histograms showing 4E-BP1-T37/46 phosphorylation levels analyzed by flow cytometry in CD4^{+} T cells, CD8^{+} T cells, and CD3-negative (CD3^{-}) cells from the spleens of

Depdc5^{ncl} and *Depdc5*^{tko} mice.

c Histogram showing the sizes of $CD8^+$ T cells (the FSC-A levels analyzed by flow cytometry) from *Depdc5*^{ncl} and *Depdc5*^{tko} mice.

d Representative histograms showing phosphorylated S6K-T389 levels in splenic $CD8^+$ T cells from *Depdc5*^{ncl} and *Depdc5*^{tko} mice after rapamycin treatment. Splenocytes obtained from *Depdc5*^{ncl} and *Depdc5*^{tko} mice treated daily with vehicle only or with 100 μg/kg rapamycin for 4 weeks then stained with fixable viability dye and anti-mouse antibodies against CD8 and S6K-T389 phosphorylation prior to analysis by flow cytometry.

e Summary bar graph showing MFI of phosphorylated S6K-T389 in splenic $CD8^+$ T cells from *Depdc5*^{ncl} and *Depdc5*^{tko} mice (n = 3).

f-h Representative flow cytometry plots (**f**) and summary bar graphs showing percentage of $CD8^+$ T cells (**g**) and $CD4^+$ T cells (**h**) in spleen from *Depdc5*^{ncl} and *Depdc5*^{tko} mice treated as described in panel (**d**).

i-k Representative flow cytometry plots (**i**) and summary bar graphs showing percentage of $CD8^+$ T cells (**j**) and $CD4^+$ T cells (**k**) in blood from *Depdc5*^{ncl} and *Depdc5*^{tko} mice treated as in panel (**d**). Error bars indicate mean ± SEM (NS, not significant, * $P < 0.05$, ** $P < 0.01$ by unpaired Student's t test).

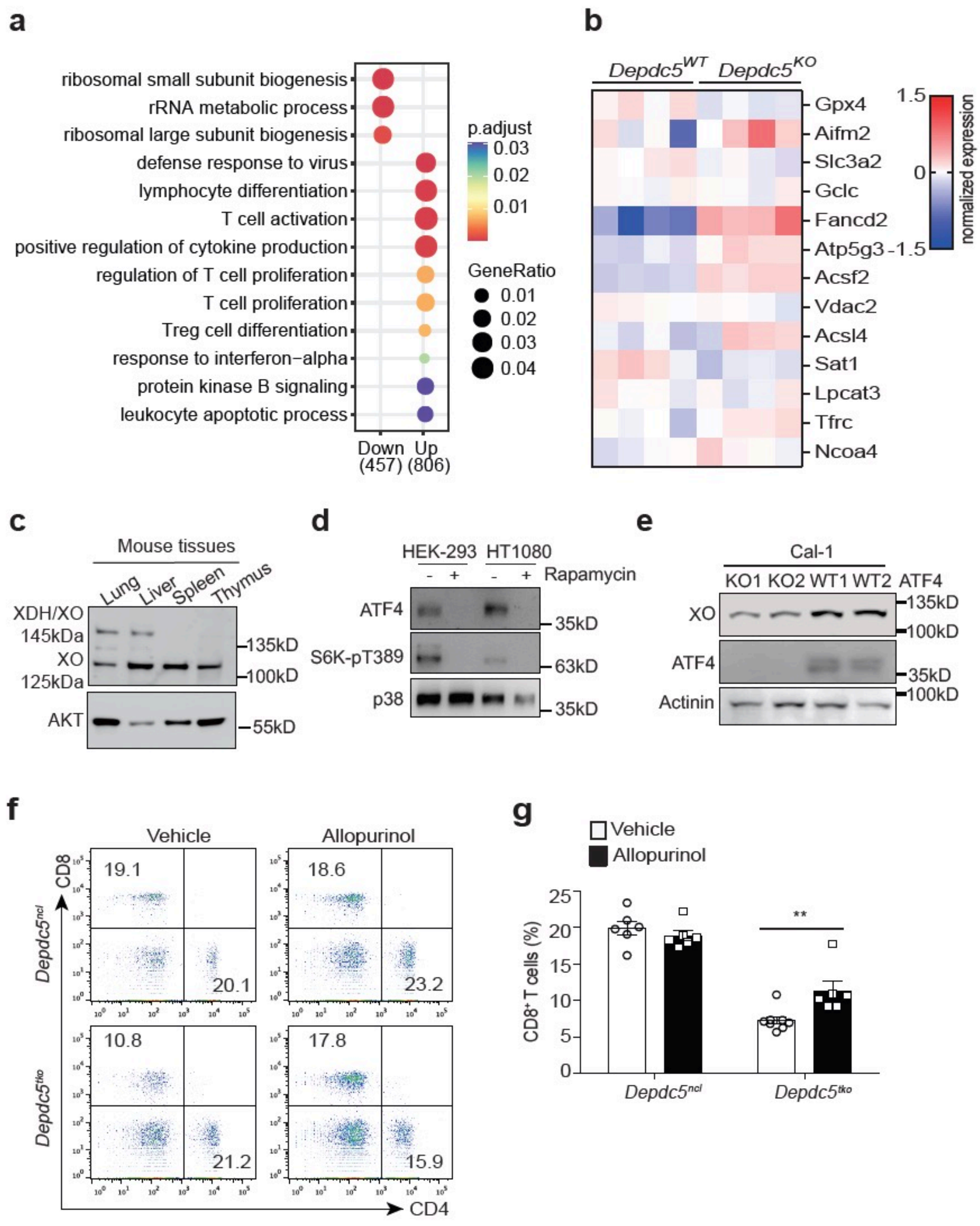

Fig.S6 DEPDC5 regulates xanthine oxidase expression via the mTORC1-ATF4 axis.

a Gene ontology (GO) enrichment analysis showing down-regulated and up-regulated biological process terms in *Depdc5* WT and *Depdc5* KO splenic CD8$^+$ T cells (*Depdc5^{KO}* vs *Depdc5^{WT}*). GO enrichment was performed using the R package cluster profiler.

b Heatmap showing normalized expression of genes associated with ferroptosis in *Depdc5^{WT}* and *Depdc5^{KO}* CD8$^+$ T cells (n = 4).

c Anti-XOR antibody immunoblotting of lung, liver, spleen and thymus tissues from wild type mice. Anti-AKT immunoblotting served as a loading control.

d Immunoblotting with antibodies against ATF4 protein and phosphorylated S6K-T389

level in HEK-293 and HT1080 cells treated with rapamycin. Anti-p38 antibody served as a control.

e Immunoblotting of XO protein level in WT and ATF4-knockout Cal-1 cells. KO1 and KO2 are two clones of ATF4-knockout Cal-1 cells. WT1 and WT2 are two clones of wild type Cal-1 cells. Actinin served as the loading control.

f Splenocytes from *Depdc5*^{ncl} and *Depdc5*^{tko} mice treated daily with vehicle or 10 mg/kg allopurinol for 4 weeks were stained with fixable viability dye and antibodies against CD4 and CD8 prior to analysis by flow cytometry. Numbers in quadrants of each plot show percentage of the gated lymphocyte subsets.

g Summary bar graphs showing percentages of CD8$^+$ T cells in spleen from *Depdc5*^{ncl} and *Depdc5*^{tko} mice treated as described in panel (**f**). Error bars indicate mean ± SEM (** $P < 0.01$ by unpaired Student's *t* test).

KEY RESOURCES TABLE

Reagent or Resource	Source	Identifier
**Antibodies**		
FITC anti-mouse CD3 Antibody (17A2)	Biolegend	Cat# 100204
Brilliant Violet 421™ anti-mouse CD4 Antibody (GK1.5)	Biolegend	Cat# 100438
Brilliant Violet 421™ anti-mouse CD8a Antibody (53-6.7)	Biolegend	Cat# 100753
APC-eFluor 780 anti-mouse CD8a Antibody (53-6.7)	eBioscience	Cat# 47-0081-82
APC-eFluor 780 anti-mouse CD4 Antibody (GK1.5)	eBioscience	Cat# 47-0041-82
PE Anti-CD45R (B220) Antibody (RA3-6B2)	eBioscience	Cat# 12-0452-82
Alexa Fluor® 647 anti-mouse CD19 Antibody (6D5)	Biolegend	Cat# 115522
Phospho-p70 S6 Kinase (Thr389) (108D2) Antibody	Cell Signaling Technology	Cat# 9234
Phospho-S6 Ribosomal Protein (Ser235/236) Antibody	Cell Signaling Technology	Cat# 2211
Phospho-4E-BP1 (Thr37/46) (236B4) Rabbit Antibody	Cell Signaling Technology	Cat# 2855
p70 S6 Kinase Antibody	Cell Signaling Technology	Cat# 9202
ATF-4 (D4B8) Rabbit Antibody	Cell Signaling Technology	Cat# 11815
p44/42 MAPK (Erk1/2) Antibody	Cell Signaling Technology	Cat# 9102
p38 MAPK (D13E1) Antibody	Cell Signaling Technology	Cat# 8690
α-Actinin (D6F6) Antibody	Cell Signaling Technology	Cat# 6487
FLAG M2 antibody	Sigma-Aldrich	Cat# F1804
Anti-rabbit IgG, HRP-linked Antibody	Cell Signaling Technology	Cat# 7074
Anti-mouse IgG, HRP-linked Antibody	Cell Signaling Technology	Cat# 7076
XDH Antibody	Proteintech	Cat# 55156-1-AP
Hsp90 Rabbit Antibody	ABclonal	Cat# A5027
Goat Anti-Rabbit IgG H&L (Alexa Fluor® 488)	Abcam	Cat# ab150081
Goat Anti-Rabbit IgG H&L (Alexa Fluor® 647)	Abcam	Cat# ab150087
InVivoMAb anti-mouse CD8α Antibody	Bio Xcell	Cat#BE0061
InVivoMAb anti-mouse PD-1 Antibody	bio Xcell	Cat#BE0146
PE mouse Anti-Ki-67	BD	Cat# 556027
**Reagents**		
Fixable Viability Stain 700	BD	Cat# 564997
Fixable Viability Stain 620	BD	Cat# 564996
BD Phosflow Fix Buffer I	BD	Cat# 557870
Perm/Wash Buffer I	BD	Cat# 557885
Sybr Green PCR Master Mix	Thermo Fisher Scientific	Cat# 4309155
Fetal bovine serum	Thermo Fisher Scientific	Cat# 10437028
C11 Bodipy^{TM} 581/591	Thermo Fisher Scientific	Cat# D3861
CM-H2DCFDA	Thermo Fisher Scientific	Cat# C6827
RPMI Medium 1640	Gibco	Cat# 11875-093
Penicillin-Streptomycin Solution, 100X	Beyotime Biotechnology	Cat# C0222
Dulbecco's Modified Eagle's Medium	CORNING	Cat# 15-017-CV
Cellular Glutathione Detection Assay Kit	Cell Signaling Technology	Cat# 13859
Deferoxamine	Sigma-Aldrich	Cat# D9533
Rapamycin	Selleck	Cat# S1039

Erastin	Selleck	Cat# S7242
Z-VAD-FMK	Selleck	Cat# S7023
Necrostatin-1	Selleck	Cat# S8037
Allopurinol	Selleck	Cat# S1630
Ferrostatin-1	Selleck	Cat# S7243
Liproxstatin-1	Selleck	Cat# S7699
**Mice**		
Tg(*Cd4*-cre)1Cwi-Chr 3	The Jackson Laboratory	Cat#017336
Depdc5$^{f/f}$ mice	This paper	N/A
**Primers**		
Cd4-cre Common: GTTCTTTGTATATGAATGTTAGCC	This paper	N/A
Cd4-cre Wild type Reverse: TATGCTCTAAGGACAAGAATTGACA	This paper	N/A
Cd4-cre Mutant Reverse: CTTTGCAGAGGGCTAACAGC	This paper	N/A
Xdh-F:ATGACGAGGACAACGGTAGAT	This paper	N/A
Xdh-R: TCATACTTGGAGATCATCACGGT	This paper	N/A
Mki67-F: CAAGGCGAGCCTCAAGAGATA	This paper	N/A
Mki67-R: TGTGCTGTTCTACATGCCCTG	This paper	N/A
mGAPDH-F: GGTGAAGGTCGGTGTGAACG	This paper	N/A
mGAPDH-R: CTCGCTCCTGGAAGATGGTG	This paper	N/A
mHmgcs1-F: AACTGGTGCAGAAATCTCTAGC	This paper	N/A
mHmgcs1-R: GGTTGAATAGCTCAGAACTAGCC	This paper	N/A
mHmgcr-F: CTGGAATTATGAGTGCCCCAAA	This paper	N/A
mHmgcr-R: ACGACTGTACTGAAGACAAAGC	This paper	N/A
mSqle-F: ATAAGAAATGCGGGGATGTCAC	This paper	N/A
mSqle-R: ATATCCGAGAAGGCAGCGAAC	This paper	N/A
